# Antimicrobial Efficacy of 1,2,3-Triazole-Incorporated Indole-Pyrazolone against Drug-Resistant ESKAPE Pathogens: Design and Synthesis

**DOI:** 10.1021/acsbiomedchemau.4c00060

**Published:** 2025-01-28

**Authors:** Dipti B. Upadhyay, Jaydeep A. Mokariya, Paras J. Patel, Subham G. Patel, Mehul P. Parmar, Disha P. Vala, Febe Ferro, Dhanji P. Rajani, Mahesh Narayan, Jyotish Kumar, Sourav Banerjee, Hitendra M. Patel

**Affiliations:** a Department of Chemistry, Sardar Patel University, Vallabh Vidyanagar, Gujarat 388120, India; b Division of Cancer Research, School of Medicine, University of Dundee, Dundee DD1 9SY, U.K.; c Microcare Laboratory and Tuberculosis Diagnosis & Research Center, Surat 395003, India; d Department of Chemistry and Biochemistry, The University of Texas at El Paso, El Paso, Texas 79968, United States

**Keywords:** cycloaddition reaction, antimicrobial activity, cytotoxicity, CuAAC click chemistry approach, ESKAPE pathogens

## Abstract

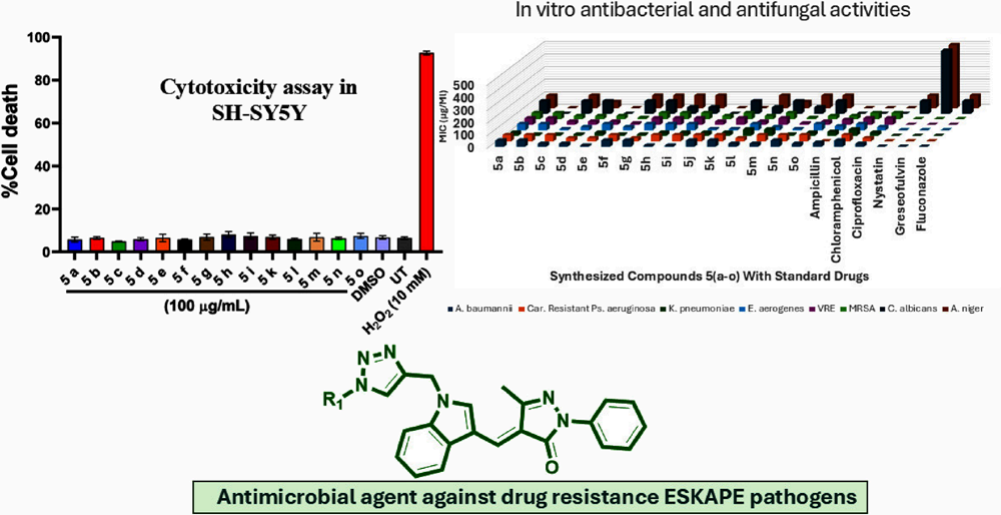

In the current study, we report the synthesis of novel 4-((1-((1*H*-1,2,3-triazole-4-yl)methyl)-1*H*-indol-3-yl)methylene)-5-methyl-2-phenyl-2,4-dihydro-3*H*-pyrazole-3-one derivatives **5a**–**o**. The compounds were prepared through a Knoevenagel condensation reaction and copper-catalyzed azide–alkyne cycloaddition (CuAAC) Click chemistry approach. The synthesized compounds exhibited promising antimicrobial activity against both Gram-positive and Gram-negative bacteria. Compounds **5e**, **5h**, and **5i** displayed potent activity with MIC value **10 μg/mL** against *Acinetobacter baumannii*, in comparison to standard drugs chloramphenicol and ampicillin. Compounds **5d**, **5h**, **5i**, **5l**, **5m,** and **5n** exhibited good-to-moderate antifungal activity against *Candida albicans* and *Aspergillus niger* equivalent to standard drugs nystatin and fluconazole. In this study, the cytotoxicity profile of a series of compounds was assessed using SHSY-5Y cells. The results indicate that compounds **5a**–**o** exhibit no significant cytotoxicity at concentrations up to **100 μg/mL**, in comparison to both untreated and vehicle control groups. These findings highlight the safety and tolerability of compounds as well as the potential of the synthesized compounds as effective agents against bacterial and fungal infections.

## Introduction

Focusing on the pressing need for new antibacterial drugs, The World Health Organization (WHO) has identified a collection of drug-resistant species (ESKAPE pathogens) that require major attention for the development of novel drugs. ESKAPE pathogens include *Enterococcus faecium* (*E. faecium*), *Staphylococcus aureus* (*S. aureus*), *Klebsiella pneumoniae* (*K. pneumoniae*), *Acinetobacter baumannii* (*A. baumannii*), *Pseudomonas aeruginosa* (*P. aeruginosa*), and *Enterobacter spp*., which pose a significant worldwide threat by causing severe and potentially life-threatening infections. Scientists are working to find new compounds to fight these antibiotic-resistant infections.^1−3^

Indole-containing nitrogen heterocyclic cores have attracted noteworthy interest as they show significant biological activities, such as anticancer, anti-inflammatory, antimicrobial, analgesic, antihypertensive, and antiatherogenic effects;^3−8^ this is because indole compounds can interact with different parts of our bodies and have an impact on various biological processes. Of particular interest in recent research has been the strategic functionalization of indole derivatives with pyrazolone and triazole moieties. Pyrazolones were prominent for their antioxidant,^9^ antimicrobial,^10,11^ antiviral,^12^ antitumor,^13^ antitubercular, analgesic,^14^ hypoglycemic,^15^ and anti-inflammatory^16^ activities, and triazoles displayed anti-inflammatory,^17^ antimicrobial,^18^ antidiabetic,^19^ antimalarial,^20^ anticancer,^21^ and antituberclulosis^22^ activities. Over the past few decades, the 1,2,3-triazole structural motif has emerged as a favored building block with a wide range of therapeutic applications.^23−25^ By integrating these distinct functional groups into the indole framework, researchers aimed to harness their synergistic effects, thus producing hybrid compounds that could potentially exhibit dual antibacterial and cytotoxicity activities.

**Figure 1:**
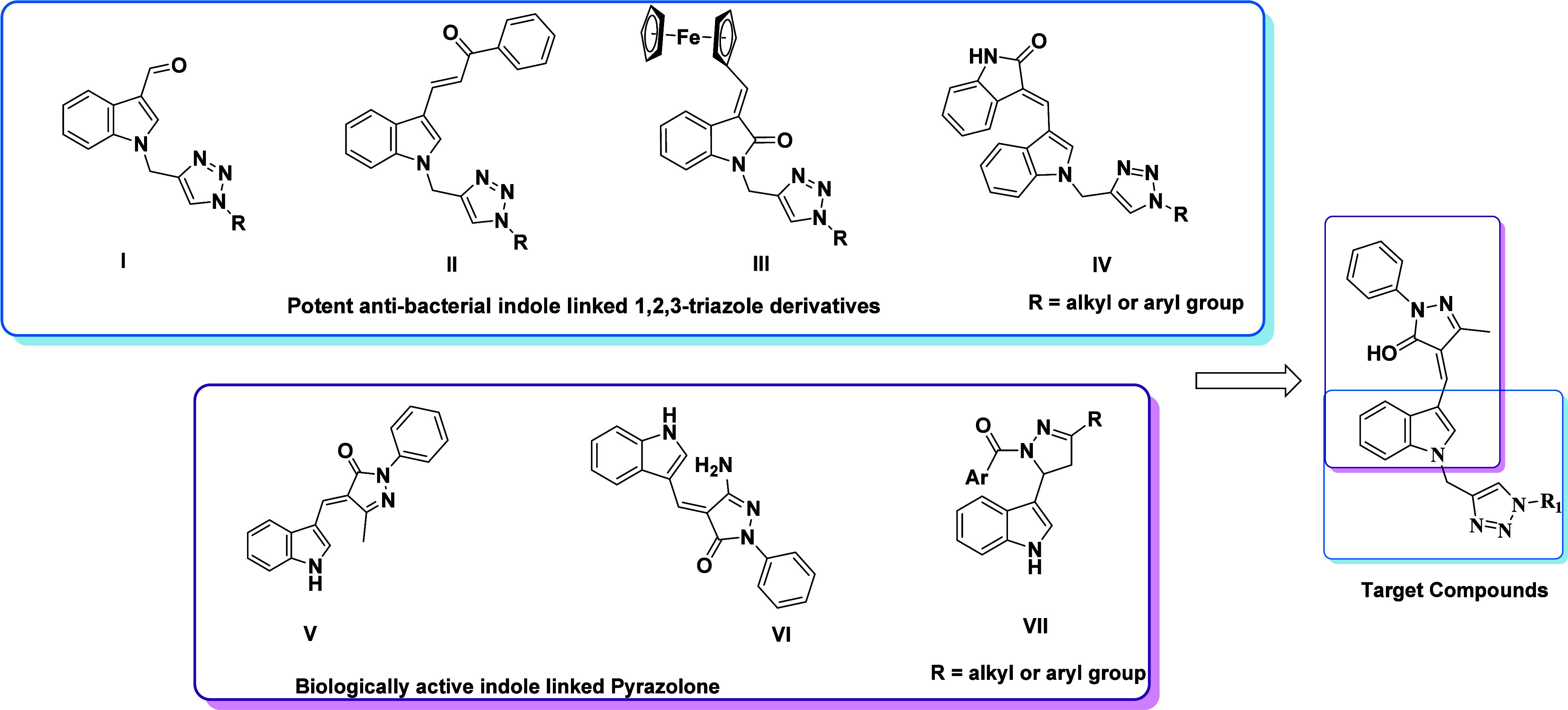
Structure design for target compounds.

Compounds based on indole-linked 1,2,3-triazole derivatives **I**–**IV** have demonstrated a wide spectrum of antimicrobial activity.^24,26−28^ Along with that, different substituted phenyl rings on the triazole exhibit various biological applications. According to the literature study, pyrazolone is a heterocyclic ring system with a potent therapeutic value that can be combined with other heterocyclic ring systems to produce a variety of biologically active entities. For instance, compounds **V**–**VII** are examples of indole and pyrazolone-containing derivatives.^29−31^ Furthermore, the combination of indole pyrazolone and indole NH-linked triazole derivatives was carried out to find potent antimicrobial profiles. Based on that, our focus in this study lies in the synthesis of novel compounds that incorporate the core structures **I**–**VII** within a single framework (Figure 1).

Cytotoxicity assessment is a crucial step in the evaluation of potential drug candidates to ensure their safety and viability for further development.^32−34^ SHSY-5Y cells are commonly employed in such studies due to their relevance to neuronal systems.^35^ This investigation is essential to determine the potential impact of these compounds on cell viability and to provide insights into their therapeutic applicability.

In this comprehensive study, we embarked on the synthesis, characterization, and biological evaluation of indole-linked pyrazolone and triazole derivatives. Based on the existing literature, the combination of indole pyrazolone and indole NH-linked triazole derivatives had not been previously explored. Therefore, in this study, we undertook the synthesis and biological evaluation of the 4-((1-((1*H*-1,2,3-triazole-4-yl)methyl)-1*H*-indol-3-yl)methylene)-5-methyl-2-phenyl-2,4-dihydro-3*H*-pyrazole-3-one derivatives. This novel approach, building upon our previous work,^36−45^ provided valuable insights into the potential therapeutic properties of such compounds and aimed to shed light on their previously unexplored synergistic effects and efficacy as potential antimicrobial agents.

The derivatives **5a**–**o** were produced via knoevenagel condensation of indole-3-carbaldehyde and 5-methyl-2-phenyl-2,4-dihydro-3*H*-pyrazole-3-one followed by the generation of 1,2,3-triazole derivatives with a very efficient Cu catalyst under ambient temperature conditions and a higher substrate scope.

## Result and Discussion

4-((1-((1*H*-1,2,3-Triazole-4-yl)methyl)-1*H*-indol-3-yl)methylene)-5-methyl-2-phenyl-2,4-dihydro-3*H*-pyrazole-3-one derivatives **5a**–**o** were synthesized through a knoevenagel condensation reaction followed by a CuAAC click approach. The process includes N-propargylation of the indole ring in the presence of propargyl bromide with K_2_CO_3_ in DMF, yielding the appropriate terminal alkyne **2a** as a white solid in 90% yield. Subsequently, the synthesis of organic azides 4**a**–**o** from the corresponding primary amine was conducted as per the previously reported route.^46^

**Scheme 1:**
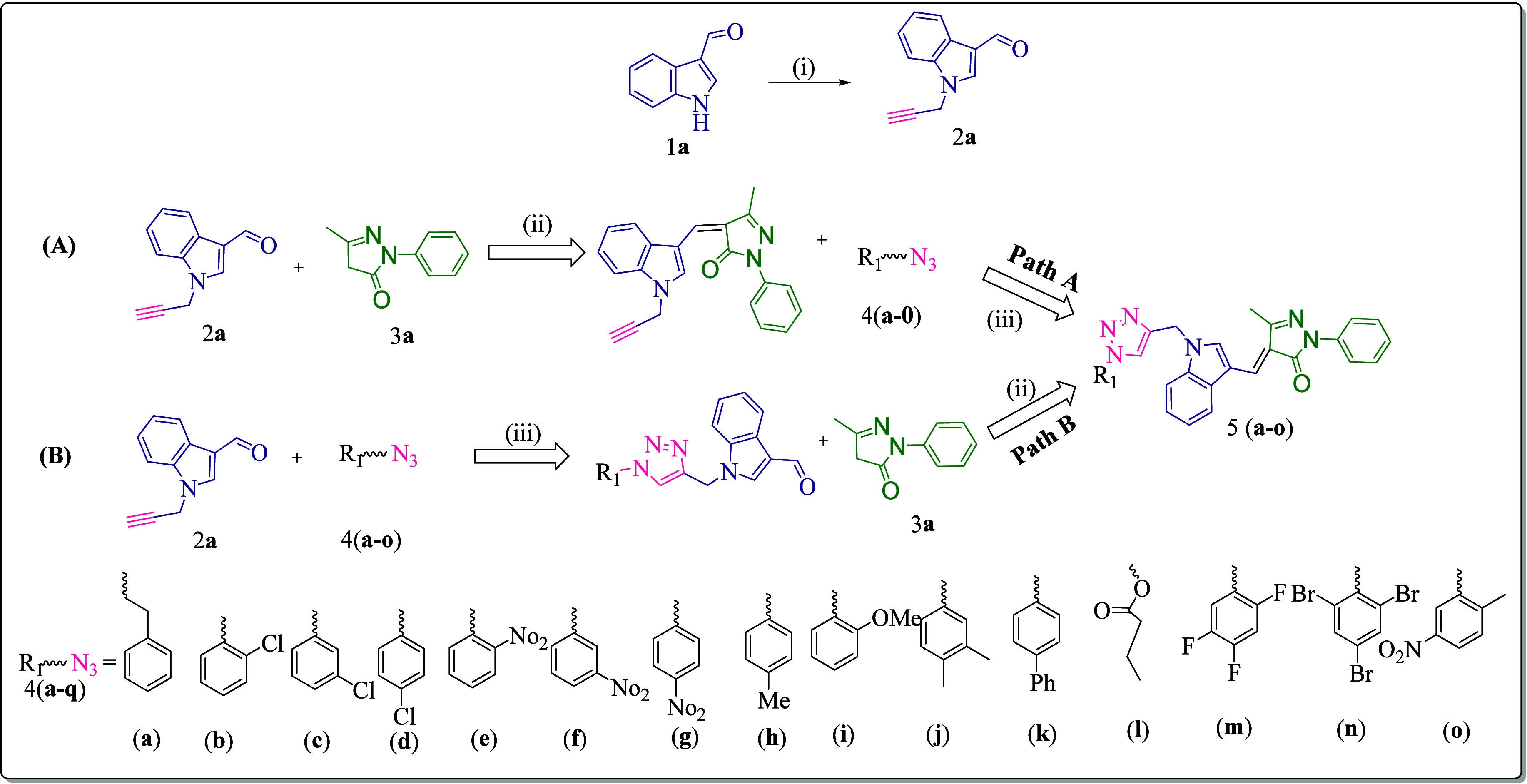
4-((1-((1*H*-1,2,3-Triazole-4-yl)methyl)-1*H*-indol-3-yl)methylene)-5-methyl-2-phenyl-2,4-dihydro-3*H*-pyrazole-3-one Derivatives **5a**–**o** (i) DMF, K_2_CO_3_, rt, 1 h, (ii) EtOH, piperidine, reflux, 30 min, (iii) CuSO_4_.5H_2_O (5 mol %), sodium ascorbate (10 mol %), DMF, water, rt, 20–30 min.

The desired target compounds **5a**–**o** were obtained in good-to-excellent yields by employing specific reaction conditions as depicted in Scheme 1. Two probable pathways were considered for the formation of compound **5a**–**o**: (i) path A involved the Knoevenagel condensation reaction of compounds **2a** and **3a** with piperidine as a base and ethanol as a solvent, followed by alkyne–azide coupling in the presence of CuSO_4_.5H_2_O and sodium ascorbate at room temperature, using DMF as the solvent. (ii) In contrast, path B consisted of an alkyne–azide coupling reaction between compounds **2a** and **4a**–**o**, followed by Knoevenagel condensation with 5-methyl-2-phenyl-2,4-dihydro-3*H*-pyrazole-3-one **3a**, leading to the formation of the desired target compounds **5a**–**o**.

The research investigated the range of substances that could undergo the reaction by using organoazides containing substituted benzyl, phenyl, and alkyl variations, following carefully optimized reaction conditions. The outcome of the reaction was notably affected by the specific substituents present on the phenyl ring, as outlined in Table 1. 1,2,3-Triazoles that have a phenyl ring with electron-withdrawing groups (4-Cl, 2-Cl, 4-Br, 4-F, and 4-NO_2_) exhibited efficient conversion to the desired product, yielding between 80 and 95%, along with a shorter reaction time. Conversely, 1,2,3-triazoles containing a phenyl ring with electron-donating groups (2-NO_2_, 3-NO_2_, 4-CH_3_, and 2,5-Di Me) resulted in lower yields and necessitated a longer reaction time for completion. The comprehensive findings are succinctly summarized in Table 1. The resulting derivatives, designated as **5a**–**o**, were characterized through ^1^H NMR, ^13^C NMR, elemental analysis, and HPLC analysis (available in the Supporting Information).

**Table 1:**
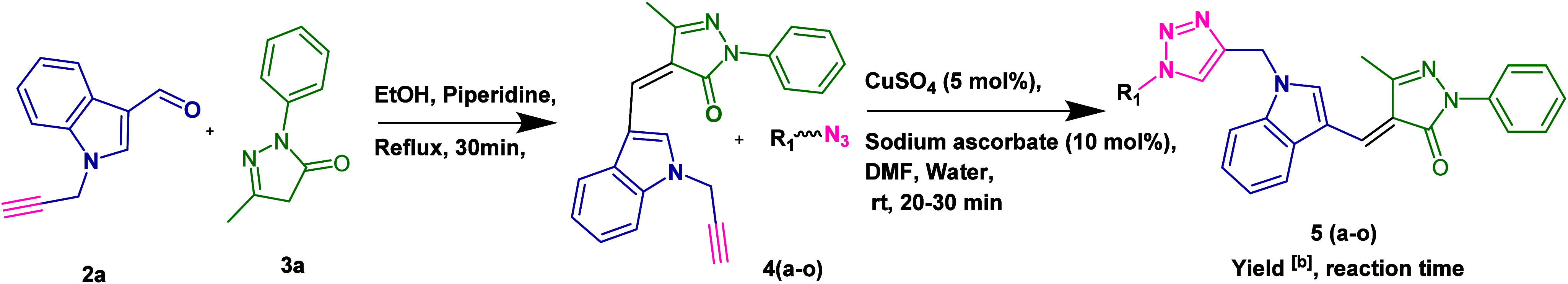

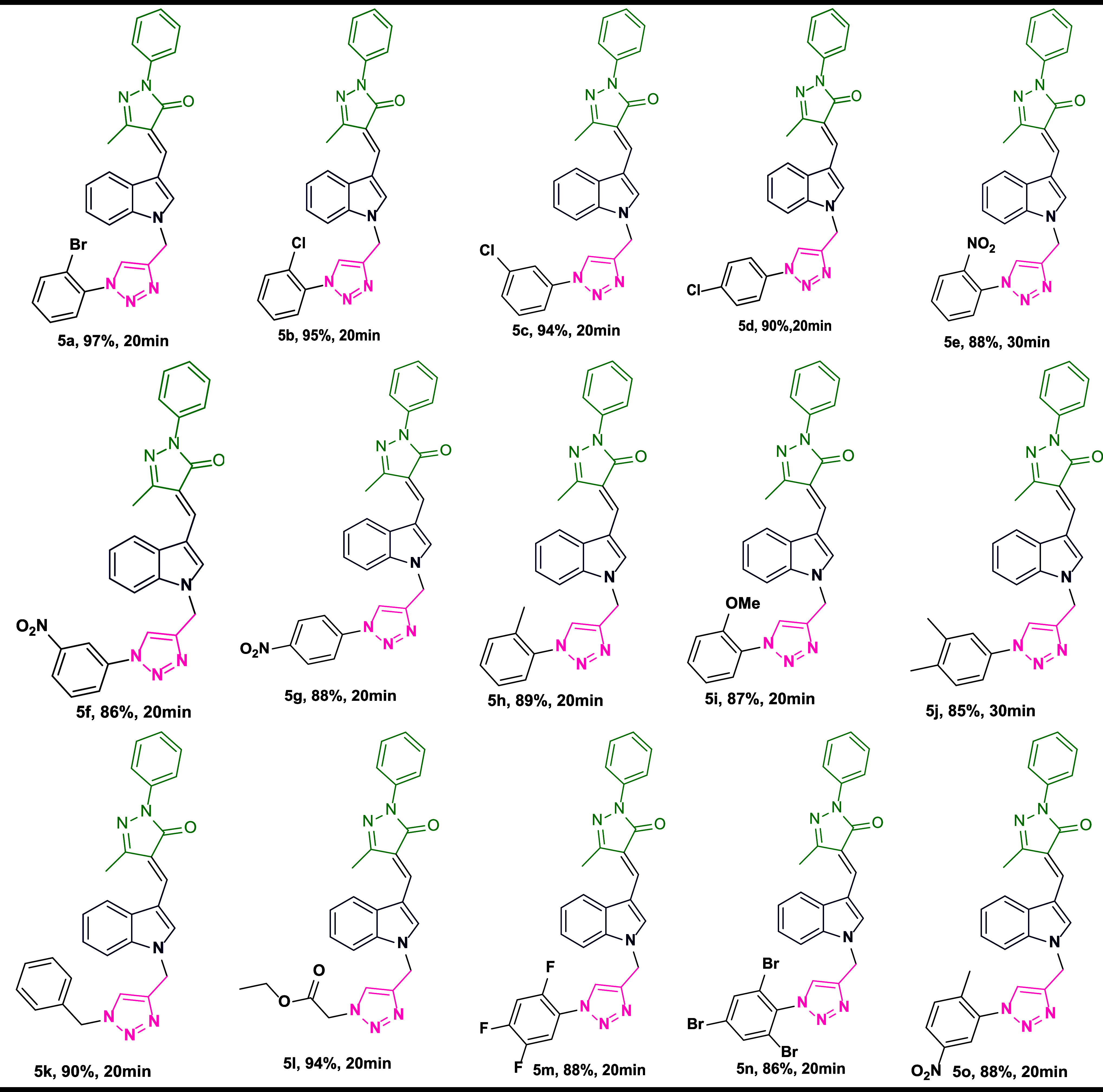
(*Z*)-4-((1-((1*H*-1,2,3-Triazole-4-yl)methyl)-1*H*-indol-3-yl)methylene)-5-methyl-2-phenyl-2,4-dihydro-3*H*-pyrazole-3-one Derivatives **5a**–**o**^a^

a Reaction condition: propargylated indole **2a**, 5-methyl-2-phenyl-2,4-dihydro-3*H*-pyrazole-3-one **3a**, and organic azide derivative **4a**–**o**, appropriate solvent and time.

b Isolated yield.

### Biological Evaluation

#### Activity against ESKAPE Pathogens

The antimicrobial assessment of the synthesized derivative **5a**–**o** was performed using the broth diffusion method. Notably, the results revealed that the synthesized compounds exhibited remarkable antimicrobial activity. Compounds **5e**, **5h**, and **5i** displayed potent efficacy with the minimum inhibitory concentrations (MIC) against *A. baumannii* of **10 μg/mL** exhibiting more potency than the standard drug chloramphenicol. Compounds **5b**, **5c**, **5d**, **5h**, **5g**, and **5n** also showed potent MIC values comparable to chloramphenicol and ampicillin, highlighting their effectiveness against this challenging Gram-negative pathogen. Compounds **5**c, **5j**, **5k**, and **5n** also showed significant action against *Enterobacter aerogenes* (*E. aerogenes*), with an MIC value of **10 μg/mL** in comparison with standard drugs. Compounds **5f** and **5n** displayed noteworthy activity against Gram-positive bacteria, including methicillin-resistant *S. aureus* and vancomycin-resistant *E. faecium*, when compared to standard drugs such as chloramphenicol, ciprofloxacin, and ampicillin.

In addition to these remarkable findings, the remaining compounds in the study also exhibited good-to-moderate antimicrobial activity against both Gram-positive and Gram-negative bacteria, with MIC values ranging from **25 to 50 μg/mL**. Overall, the screening results underscore the promising antimicrobial potential of these synthesized compounds, particularly in the context of addressing infections caused by ESKAPE pathogens.

#### *In Vitro* Antifungal Activity

The screening results of antifungal activity for the synthesized compounds **5a**–**o** were found to be active for most of the compounds against the standard drug mentioned in Table 2. Specifically, compounds **5d**, **5h**, **5i**, **5l**, **5m**, and **5n** displayed potent activity against *Candida albicans* (*C. albicans*) and *Aspergillus niger* (*A. niger*), surprassing the standard drug greseofulvin and fluconazole with MIC values of **50 μg/mL**. Furthermore, the remaining compound also exhibited good activity with MIC values ranging from **100** to **>100 μg/mL** against the standard drugs nystatin, greseofulvin, and fluconazole (Table 2).

**Table 2:**
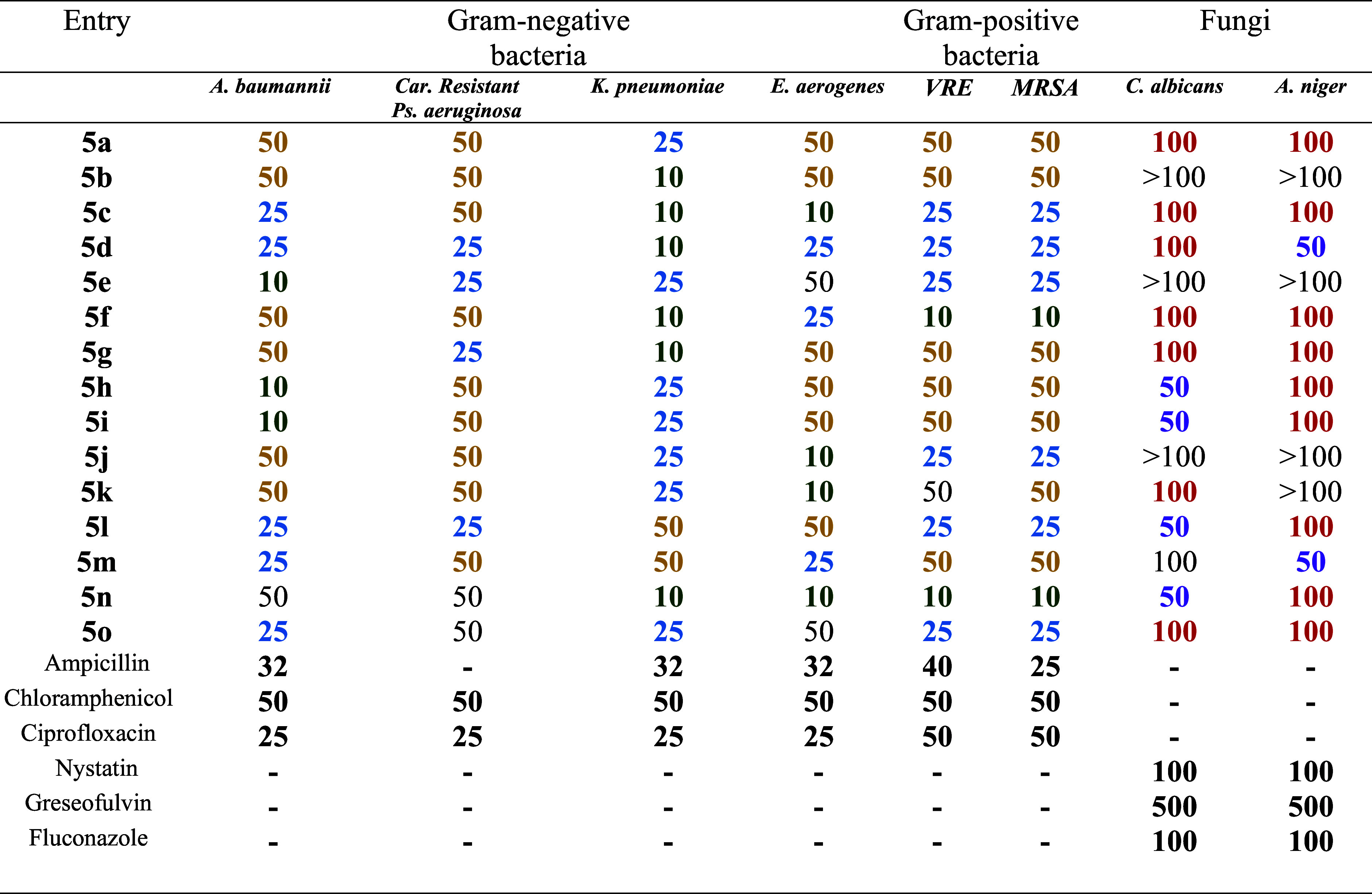
*In Vitro* Antibacterial and Antifungal Activities [MIC (μg/mL)] of the Synthesized Compounds **5a**–**q**^a^

a **Bold and green** values show more potency as compared to ampicillin and ciprofloxacin. **Bold and blue** values show more potency as compared to ampicillin and ciprofloxacin. **Bold and yellow** values show more potency as compared to Chloramphenicol. **Bold and purple** values show more potency as compared to nystatin. **Bold and red** values show more potency as compared to griseofulvin and fluconazole. **- not determined. Abbreviation**:*A. baumannii*:*Acinetobacter baumannii*,carbapenem resistance *Pseudomonas aeruginosa*,*Klebsiella pneumoniae*,*Enterobacter aerogenes*, ***VRE***: vancomycin-resistant enterococcus,***MRSA***: methicillin-resistant *Staphylococcus aureus*,*Candida albicans*,*Aspergillus niger*.

#### *In Vitro* Bactericidal Activity

Following the promising results of the MIC study, these synthesized derivatives **5a**–**o** were further evaluated for their *in vitro* bactericidal activity. Table 3 provides a comprehensive overview of the *in vitro* bactericidal activity (expressed as the minimum bactericidal concentration, MBC, in μg/mL) of synthesized compounds **5a**–**o** against various Gram-negative and Gram-positive bacteria strains, including multidrug-resistant pathogens. Notably, compounds **5b**, **5c**, **5d**, **5e, 5f**, **5g**, **5h**, and **5n** exhibit potent activity, surpassing that of established antibiotics such as ampicillin, ciprofloxacin, and chloramphenicol. The highlighted values indicate superior efficacy against specific bacterial strains, demonstrating potential for combating antibiotic-resistant infections. These findings underscore the significance of these compounds as promising candidates for further *in vivo* evaluation

**Table 3:**
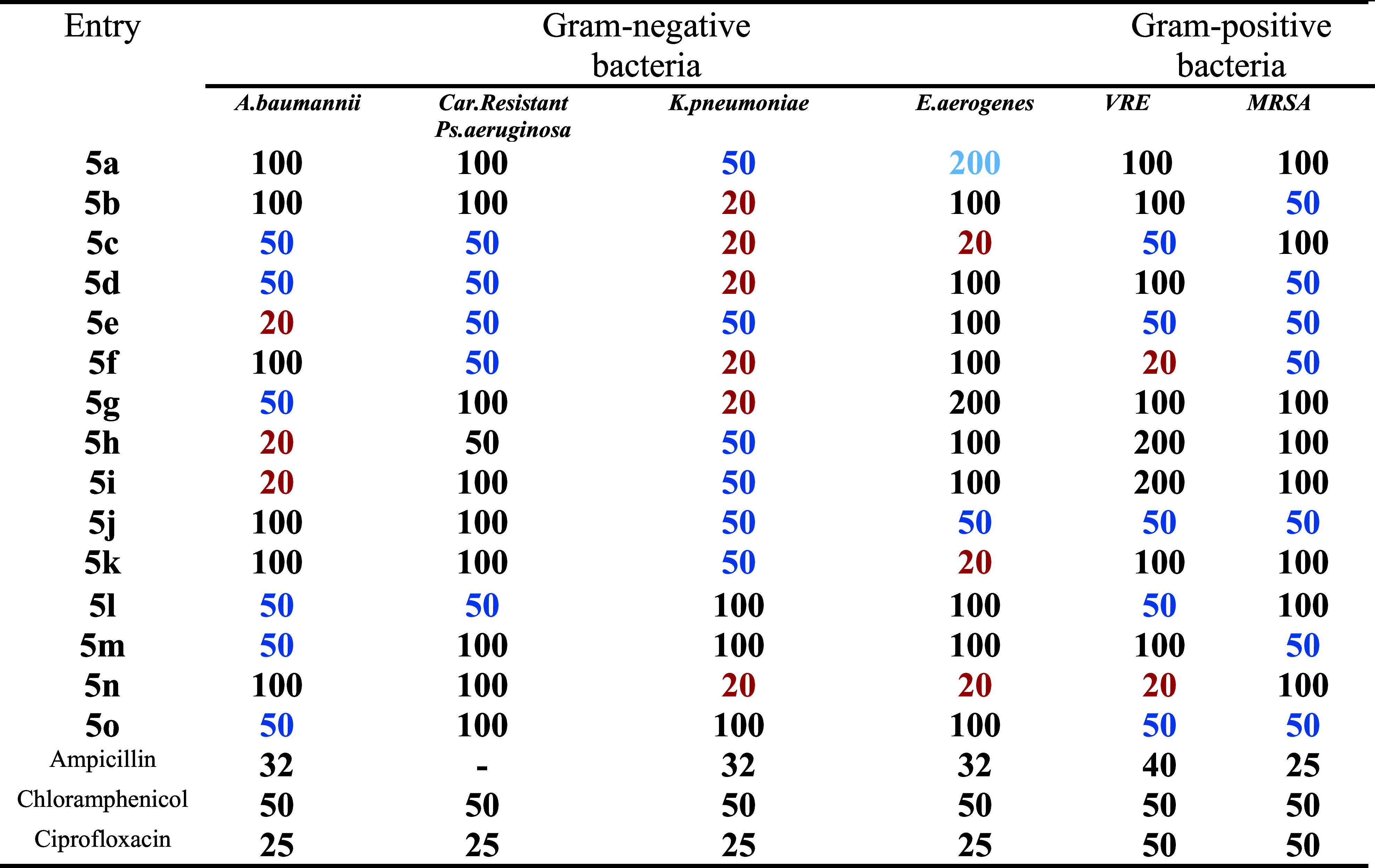
*In Vitro* Bactericidal Activity [MBC (μg/mL)] of the Synthesized Compounds **5a**–**o**^a^

a **Bold and red** values show more potency as compared to ampicillin and ciprofloxacin. **Bold and blue** values show equivalent potency as compared to Chloramphenicol. **Abbreviation:***A. baumannii*: *Acinetobacter baumannii*, ***Car. Resistant****P. aeruginosa*:carbapenem-resistant *Pseudomonas aeruginosa*, *K. pneumoniae*:*Klebsiella pneumoniae*, *E. aerogenes*: *Enterobacter aerogenes*, *VRE*:vancomycin-resistant enterococcus, ***MRSA***:methicillin-resistant *Staphylococcus aureus*, *C. albicans*:*Candida albicans*, *A. niger*: *Aspergillus niger*.

.

#### *In Vitro* Cytotoxicity Measurement and Confocal Imaging of Compounds Using Hoechst and Propidium Iodide

**Figure 2:**
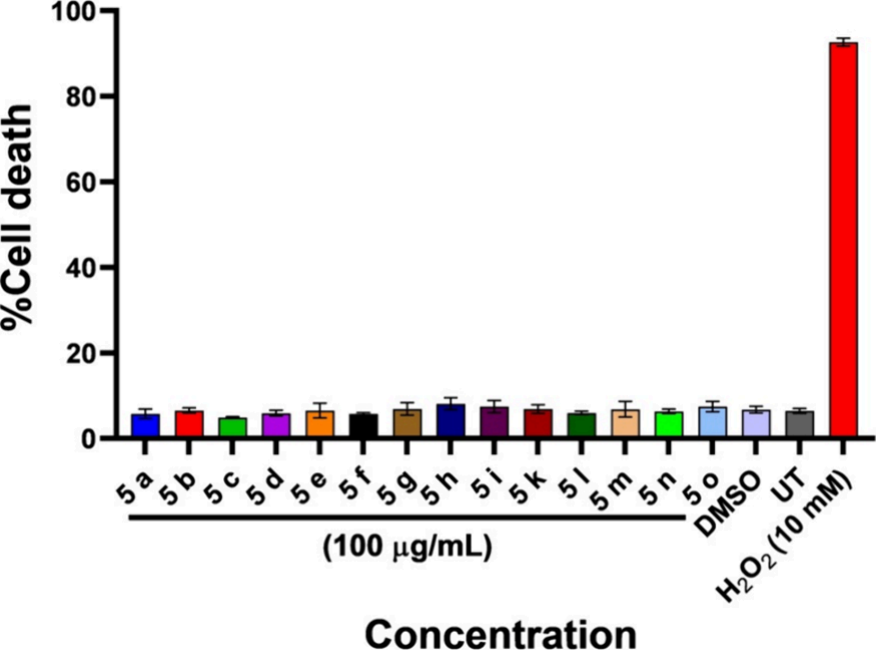
Cytotoxicity assay in a human neuroblastoma cell line (SH-SY5Y) using Hoechst (H) and propidium iodide at drug concentrations of **100 μg/mL**. Graphical data indicate averages of triplicates of results from cytotoxicity measurements with standard deviation values (±SD) represented in each bar.

Cytotoxicity assessment using SHSY-5Y cells as shown in Figure 2 yielded significant findings, particularly concerning compounds **5a**–**o**. Notably, even at **100 μg/mL**, these compounds exhibited no cytotoxic effects in comparison to both untreated and vehicle control groups (Figure 2), implying a promising safety profile for these compounds. This absence of cytotoxicity at these concentrations suggests a potential therapeutic advantage, allowing for benefits without compromising the cell viability. The compounds having a safety profile encourage their consideration for application in neuronal-related disorders, as they demonstrate noncytotoxic behavior even at physiological levels.

The cytotoxicity assay underscores the promising safety of compounds **5a**–**o**. Their lack of cytotoxic effects, even at higher concentrations, points toward their potential in therapeutics. These findings highlight these compounds as viable candidates for addressing neuronal disorders, emphasizing their significance in advancing therapeutic strategies.

**Figure 3:**
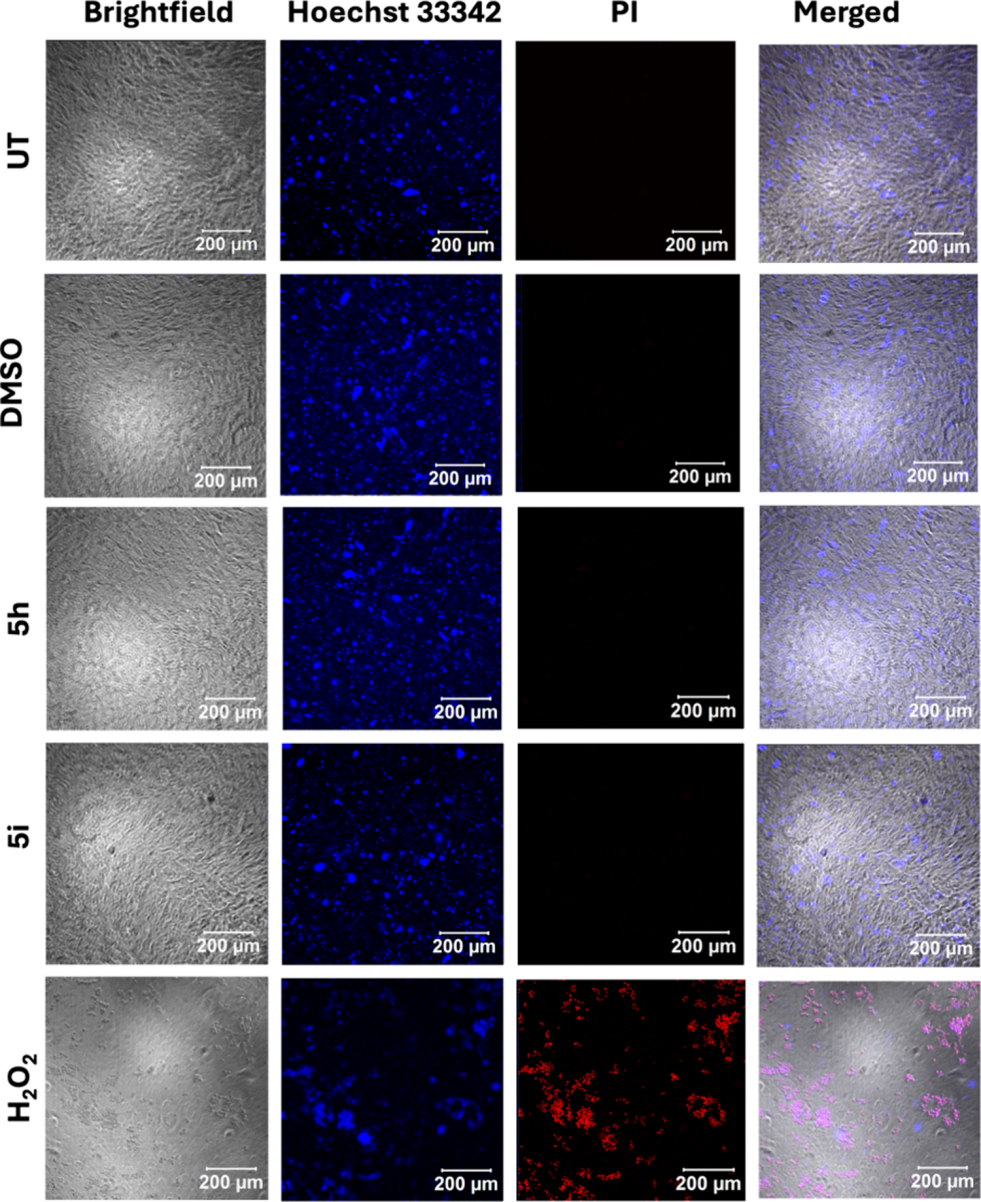
Confocal microscopic images of SH-SY5Y cells using Hoechst and PI in the untreated control, DMSO-treated cells, H_2_O_2_ (positive control), and cells treated with **100 μg/mL** of compounds **5h** or **5i** (higher cytotoxicity than other compounds).

Cytotoxic activities of the compounds further validated by confocal live-cell imaging Live cells were identified using Hoechst and propidium iodide with H_2_O_2_-treated cells as a positive control. Control DMSO-treated and compound (**5a**–**o**)-treated cells were compared, and only a few or no dead cells were observed in compound-treated cells; two of them (comparatively higher cell death) are represented in Figure 3.

## Conclusions

In response to the surge in drug-resistant microbial strains, this study presents a synthesis of a series of synthesized derivatives **5a**–**o**. The synthesized compounds were subjected to thorough biological evaluation, revealing promising antimicrobial activity against both Gram-positive and Gram-negative bacteria. Notably, compounds **5e**, **5h**, and **5i** demonstrated excellent efficacy with a MIC value of **10 μg/mL** against *A. baumannii*, a challenging Gram-negative pathogen. Additionally, compounds **5d**, **5h**, **5i**, **5l**, **5m**, and **5n** exhibited potent antifungal activity against *C. albicans* and *A. niger*, outperforming standard drugs with an MIC value of **50 μg/mL**. The collective results of this study underscore the potential of these synthesized compounds as valuable candidates for the development of new antimicrobial agents. The cytotoxicity assessment of compound **5a**–**o** using SHSY-5Y cells demonstrates their noncytotoxic nature up to **100 μg/mL** concentration. These findings open avenues for continued research, emphasizing the importance of further studies to elucidate the therapeutic potential and mechanisms underlying the observed safety profile of these compounds.

## Experimental Section

### General Methods

All chemicals were purchased from commercially available sources and used without further purification. The progress of all chemical reactions was monitored by thin-layer chromatography (TLC, on aluminum plates precoated with F254 silica gel 60). Melting points of all solid compounds were determined by the open capillary tube method and are uncorrected. Nuclear magnetic resonance NMR spectra were recorded on Bruker AVIII-400 MHz NMR or Bruker AVIII-500 MHz spectrometers at 298 K. A residual protio solvent was used as a reference for ^1^H spectra in deuterated solvent samples. Abbreviations used for the ^1^H NMR signal are as follows: s = singlet, d = doublet, t = triplet, q = quartet dd = double doublet, and m = multiplet. The chemical shifts are reported in parts per million, and coupling constants (*J*) are provided in Hertz.

#### General Procedure for the Synthesis of 1-(Prop-2-yn-1-yl)-1*H*-indole-3-carbaldehyde (**2a**)

A 50 mL round-bottom flask was charged with 1*H*-indole-3-carbaldehyde (1 mmol), propargyl bromide (1 mmol), and anhydrous potassium carbonate (3 mmol). Dimethylformamide (5 mL) was added as the solvent, and the flask was equipped with a mechanical stirrer. The reaction mixture was stirred for 1 h at room temperature, and the progress of the reaction was monitored by thin-layer chromatography (TLC). After completion of the reaction, the reaction mixture was poured into a separate container with 50 mL of ice-cold water while continuously stirring to quench the reaction and induce precipitation. A dilute hydrochloric acid (HCl) solution was added to the precipitates until the pH of the mixture reached neutral (around pH 7) to neutralize the basic species. The resulting mixture was filtered to separate the precipitates of 1-(prop-2-yn-1-yl)-1*H*-indole-3-carbaldehyde (**2a**), washed with water, dried, and then recrystallized from ethanol.

#### General Procedure for the Synthesis of 4-((1-((1*H*-1,2,3-Triazole-4-yl)methyl)-1*H*-indol-3-yl)methylene)-5-methyl-2-phenyl-2,4-dihydro-3*H*-pyrazole-3-one Derivatives **5a**–**o**

##### Path A

In this procedure, a mixture of 1 mmol of 1-(prop-2-yn-1-yl)-1*H*-indole-3-carbaldehyde **2a** and 1 mmol of 5-methyl-2-phenyl-2,4-dihydro-3*H*-pyrazole-3-one **3a**, along with the catalytic amount of piperidine (2 mL) and 3 mL of ethanol, was introduced into a round-bottom flask. After refluxing for 2 h and monitoring the reaction by TLC, a solid mass formed in the flask. The resulting mixture was filtered, washed in ethanol, and dried. To this, 1.5 mmol of azide **4a**–**q**, copper sulfate pentahydrate (5 mol %), and sodium ascorbate (10 mol %) were added in DMF (3 mL) and vigorously stirred for 10–30 min while tracking the progress with TLC. Upon completion, the reaction mixture was poured into crushed ice, and the resulting solid mass **5a**–**o** was filtered and dried.

##### Path B

In this process, a mixture of 1 mmol of 1-(prop-2-yn-1-yl)-1*H*-indole-3-carbaldehyde **2a** and 1.5 mmol of azide **4a**–**q**, along with copper sulfate pentahydrate (5 mol %) and sodium ascorbate (10 mol %) in DMF (3 mL), underwent rigorous stirring for 10–30 min. The reaction progress was tracked by TLC. After completion, the mixture was poured onto crushed ice, filtered, and dried. Subsequently, a blend of 1 mmol of 1-(prop-2-yn-1-yl)-1*H*-indole-3-carbaldehyde **2a** and 1 mmol of 5-methyl-2-phenyl-2,4-dihydro-3*H*-pyrazole-3-one **3a**, along with catalytic piperidine (2 mL) and 3 mL of ethanol, was refluxed for 2 h with TLC monitoring. After the reaction, solid mass formation prompted filtration, ethanol washing, and drying for mixture **5a**–**o**. Then, a mixture of azide 8 (1 mmol), terminal alkyne 7 (1 mmol), copper sulfate pentahydrate (5 mol %), and sodium ascorbate (10 mol %) in DMF (3 mL) was stirred for 10–30 min and monitored by TLC. After completion, the mixture was quenched in crushed ice, filtered, and dried.

#### 4-((1-((1-(2-Bromobenzyl)-1*H*-1,2,3-triazol-4-yl)methyl)-1*H*-indol-3-yl)methylene)-5-methyl-2-phenyl-2,4-dihydro-3*H*-pyrazol-3-one (**5a**)

Yellow solid (0.390 g, 97%), mp: 178–180 °C; ^1^H NMR (500 MHz, DMSO-*d*_6_) δ: 9.90 (s, 1H, CH–N), 8.32 (s, 1H, Ar–H), 8.22–8.20 (m, 1H, Ar–H), 8.09 (s, 1H, Ar–H), 7.83–7.80 (m, 1H, Ar–H), 7.54 (dd, *j* = 2 and 12 Hz, 2H, Ar–H), 7.45 (dt, *j* = 2 and 16 Hz, 2H, Ar–H), 7.37 (dt, *j* = 2 and 11 Hz, 2H, Ar–H), 7.25 (dd, *j* = 2 and 14 Hz, 2h, Ar–H), 7.19 (dt, *j* = 1 and 15.5 Hz, 1H, Ar–H), 5.74 (s, 2H, CH_2_), 5.58 (s, 2H, CH_2_), 2.43 (s, 3H, CH_3_). ^13^C NMR (126 MHz, DMSO) δ 13.51 (CH3), 42.54 (CH2), 52.57 (CH2), 112.14 (CH), 112.33 (Ar–C), 113.46 (Ar–C), 118.59 (Ar–C), 119.21 (Ar–C), 119.44 (Ar–C), 121.90 (C–N), 123.07 (Ar–C), 124.19 (Ar–C), 124.55 (Ar–C), 124.70 (Ar–C), 129.26 (Ar–C), 129.31 (Ar–C), 130.64 (C–N), 135.75 (Ar–C), 136.76 (Ar–C), 137.05 (Ar–C), 139.28 (Ar–C), 140.68 (Ar–C), 142.78 (CH), 151.49 (C–N), 163.23 (C=O). *Anal*. Calcd for C_29_H_23_BrN_6_O: C, 63.16; H, 4.20; Br, 14.49; N, 15.24; O, 2.90%; Found: C, 63.41; H, 4.19; N, 15.30; O, 2.91%. MS (ESI-TOF) *m*/*z* calcd. for C_29_H_24_BrN_6_O (M+H)+: 551.12, found: 551.20, HPLC purity: 99.10%.

#### 4-((1-((1-(2-Chlorophenyl)-1*H*-1,2,3-triazol-4-yl)methyl)-1*H*-indol-3-yl)methylene)-5-methyl-2-phenyl-2,4-dihydro-3*H*-pyrazol-3-one (**5b**)

Yellow solid (0.414 g, 95%), mp: 180–200 °C; ^1^H NMR (500 MHz, DMSO) δ 9.96 (s, 1H, CH–N), 8.81 (s, 1H, Ar–H), 8.23 (dd, *j =* 2.5 and 8.5 Hz, 1H, Ar–H), 8.15 (s, 1H, Ar–H), 7.90 (dd, *j* = 2 and 9 Hz, 1H, Ar–H), 7.78 (dd, *j* = 1 and 9 Hz, 1H, Ar–H), 7.70 (dd, *j* 2 and 9.5 Hz, 1H, Ar–H), 7.64 (dt, *j* 2 and 17.5 Hz, 1H, Ar–H), 7.58 (t, *j* 7.5 Hz, 1H, Ar–H), 7.45 (t, *j* = 7.5 Hz, 2H, Ar–H), 7.40 (dt, *j* = 1.5 and 12.5 Hz, 2H, Ar–H), 7.18 (t, *j* = 7.5 Hz, 1H, Ar–H), 5.86 (s, 2H, CH_2_), 2.41 (s, 3H, CH_3_); ^13^C NMR (126 MHz, DMSO) δ 163.24, 151.51, 140.72, 139.29, 137.08, 136.83, 132.26, 131.03, 129.36, 129.24, 128.93, 128.85, 126.95, 124.53, 124.23, 123.11, 119.46, 119.28, 118.54, 112.42, 112.18, 42.36, 13.51. Anal. Calcd for C_28_H_21_ClN_6_O: C, 68.22; H, 4.29; Cl, 7.19; N, 17.05; O, 3.25%; Found: C, 68.42; H, 4.30; N, 17.00; and O, 3.24%. MS (ESI-TOF) *m*/*z* calcd. for C_28_H_22_ClN_6_O (M+H)+: 493.15, found: 493.09, HPLC purity: 99.69%.

#### 4-((1-((1-(3-Chlorophenyl)-1*H*-1,2,3-triazol-4-yl)methyl)-1*H*-indol-3-yl)methylene)-5-methyl-2-phenyl-2,4-dihydro-3*H*-pyrazol-3-one (**5c**)

Yellow solid (0.410 g, 94%), mp: 180–200 °C; ^1^H NMR (500 MHz, DMSO) δ 9.99 (s, 1H, CH–N), 9.06 (s, 1H, Ar–H), 8.23 (dd, *j* = 1.5 and 9.5 Hz, 1H, Ar–H), 8.12 (s, 1H, Ar–H), 8.02 (dd, *j* = 2 and 10 Hz, 3H, Ar–H), 7.90 (td, *j* = 1 and 10 Hz, 1H, Ar–H), 7.84 (dd, *j* = 2 and 8 Hz, 1H, Ar–H), 7.61 (t, *j* = 8 Hz, 1H, Ar–H), 7.56 (td, *j* = 1.5 and 11 Hz, 1H, Ar–H), 7.44 (dt, *j* = 2 and 16 Hz, 2H, Ar–H), 7.40–7.37 (m, 2H, Ar–H), 7.18 (tt, *j* = 1 and 17 Hz, 1H, Ar–H), 5.85 (s, 2H, CH_2_), 2.43 (s, 3H, CH_3_). ^13^C NMR (126 MHz, DMSO) δ 13.51 (CH3), 42.36 (CH2), 112.18 (CH), 112.42 (CH), 118.54 (Ar–C), 119.28 (C–N), 119.46 (Ar–C), 123.11 (Ar–C), 124.23 (Ar–C), 124.53 (Ar–C), 126.95 (Ar–C), 128.85 (Ar–C), 128.93 (Ar–C), 129.24 (Ar–C), 129.36 (Ar–C), 131.03 (C–N), 132.26 (Ar–C), 136.83 (Ar–C), 137.08 (Ar–C), 139.29 (Ar–C), 140.72 (CH), 151.51 (C–N), 163.24 (C=O). Anal. Calcd for C_28_H_21_ClN_6_O: C, 68.22; H, 4.29; Cl, 7.19; N, 17.05; O, 3.25%; Found: C, 68.36; H, 4.31; N, 17.00; O, 3.24%. MS (ESI-TOF) *m*/*z* calcd. for C_28_H_22_ClN_6_O (M+H)+: 493.15, found: 493.07, HPLC purity: 99.77%.

#### 4-((1-((1-(4-Chlorophenyl)-1*H*-1,2,3-triazol-4-yl)methyl)-1*H*-indol-3-yl)methylene)-5-methyl-2-phenyl-2,4-dihydro-3*H*-pyrazol-3-one (**5d**)

Yellow solid (0.391 g, 90%), mp: 178–180 °C; ^1^H NMR (500 MHz, DMSO-*d*_6_) δ: 9.99 (s, 1H, CH–N), 9.03 (s, 1H, Ar–H), 8.23 (dd, *j* = 1.5 and 9 Hz, 1H, Ar–H), 8.12 (s, 1H, Ar–H), 8.02 (dd, *j* = 1.5 Hz ans 10 Hz, 2H, Ar–H), 7.93 (dd, *j* = 2 and 9 Hz, 2H, Ar–H), 7.85 (dd, *j* = 2 and 9 Hz, 1H, Ar–H), 7.66 (dd, *j* = 2 and 12 Hz, 2H, Ar–H), 7.44 (dt, *j* = 2 and 16 Hz, 2H, Ar–H), 7.40–7.37 (m, 2H, Ar–H), 7.38 (tt, *j* = 1 and 17 Hz, 1H, Ar–H), 5.85 (s, 2H, Ar–H), 2.43 (s, 3H, CH_3_). ^13^C NMR (126 MHz, DMSO) δ 13.50 (CH3), 42.58 (CH2), 112.22 (CH2), 112.35 (Ar–C), 118.53 (Ar–C), 119.26 (C–N), 119.35 (Ar–C), 119.47 (Ar–C), 120.46 (Ar–C), 123.01 (Ar–C), 123.10 (Ar–C), 124.27 (Ar–C), 124.53 (Ar–C), 129.13 (Ar–C), 129.25 (Ar–C), 129.37 (Ar–C), 132.09 (C–N), 134.65 (Ar–C), 136.73 (Ar–C), 137.08 (Ar–C), 137.93 (Ar–C), 139.28 (Ar–C), 140.84 (Ar–C), 143.78 (CH), 151.51 (C–N), 163.24 (C=O). Anal. Calcd for C_28_H_21_ClN_6_O: C, 68.22; H, 4.29; Cl, 7.19; N, 17.05; O, 3.25%; Found: C, 68.02; H, 4.28; N, 17.10; and O, 3.24%. MS (ESI-TOF) *m*/*z* calcd. for C_28_H_22_ClN_6_O (M+H)+: 493.15, found: 493.06, HPLC purity: 99.10%.

#### 5-Methyl-4-((1-((1-(2-nitrophenyl)-1*H*-1,2,3-triazol-4-yl)methyl)-1*H*-indol-3-yl)methylene)-2-phenyl-2,4-dihydro-3*H*-pyrazol-3-one (**5e**)

Yellow solid (0.392 g, 88%), mp: 240–242 °C; ^1^H NMR (500 MHz,) δ 9.99 (s, 1H, CH–N), 9.03 (s, 1H, Ar–H), 8.23 (s, 1H, Ar–H), 8.12 (s, 1H, Ar–H), 8.03 (d, *j* = 8 Hz, 2H, Ar–H), 7.93 (t, *j* = 7.5 Hz, 1H, Ar–H), 7.86 (t, *j* = 2 Hz, 2H, Ar–H), 7.42 (td, *j* = 2.5 and 26 Hz, 4H, Ar–H), 7.19 (t, *j* = 7.5 Hz, 2H, Ar–H), 5.88 (s, 2H, CH_2_), 2.44 (s, 3H, CH_3_). ^13^C NMR (126 MHz, DMSO) δ 13.51 (CH3), 62.23 (CH2), 107.76 (CH), 112.25 (Ar–C), 118.54 (Ar–C), 119.31 (C–N), 119.47 (Ar–C), 122.37 (Ar–C), 129.25 (Ar–C), 130.33 (C–N), 133.40 (Ar–C), 135.70 (Ar–C), 140.85 (CH), 151.51 (C–N), 163.24 (C=O). Anal. Calcd for C_28_H_21_N_7_O_3_: C, 66.79; H, 4.20; N, 19.47; O, 9.53%; Found: C, 66.99; H, 4.22; N, 19.41; O, 9.50%. MS (ESI-TOF) *m*/*z* calcd. for C_28_H_22_N_7_O_3_ (M+H)+: 504.18, found: 504.09, HPLC purity: 99.74%.

#### 5-Methyl-4-((1-((1-(3-nitrophenyl)-1*H*-1,2,3-triazol-4-yl)methyl)-1*H*-indol-3-yl)methylene)-2-phenyl-2,4-dihydro-3*H*-pyrazol-3-one (**5f**)

Yellow solid (0.384 g, 86%), mp: 210–212 °C; ^1^H NMR (500 MHz,) δ: 10.00 (s, 1H, CH–N), 9.23 (s, 1H, Ar–H), 8.71 (s, 1H, Ar–H), 8.39 (dd, *j* = 2.5 and 10.5 Hz, 1H, Ar–H), 8.32 (dd, *j* = 2.5 and 10.5 Hz, 1H, Ar–H), 2.23 (dd, *j* = 2.5 and 9 Hz, 1H, Ar–H), 8.12 (s, 1H, Ar–H), 8.02 (d, *j* = 8 Hz, 2H, Ar–H), 7.87 (q, *j* = 8 Hz, 2H, Ar–H), 7.44 (t, *j* = 8 Hz, 2H, Ar–H), 7.39 (t, *j* = 6.5 Hz, 2H, Ar–H), 7.18 (t, *j* = 7 Hz, Ar–H), 5.87 (s, 2H, CH_2_), 2.43 (s, 3H, CH_3_). ^13^C NMR (126 MHz, DMSO) δ: 13.51 (CH3), 49.71 (CH2), 112.21 (CH), 112.41 (Ar–C), 118.56 (Ar–C), 119.34 (C–N), 119.45 (Ar–C), 124.54 (Ar–C), 126.03 (Ar–C), 128.18 (Ar–C), 129.25 (Ar–C), 129.36 (C–N), 134.90 (Ar–C), 137.09 (CH), 140.82 (C–N), 163.25 (C=O). Anal. Calcd for C_28_H_21_N_7_O_3_: C, 66.79; H, 4.20; N, 19.47; O, 9.53%; Found: C, 66.92; H, 4.21; N, 19.39; O, 9.49%, HPLC purity: 99.42%.

#### 5-Methyl-4-((1-((1-(4-nitrophenyl)-1*H*-1,2,3-triazol-4-yl)methyl)-1*H*-indol-3-yl)methylene)-2-phenyl-2,4-dihydro-3*H*-pyrazol-3-one (**5g**)

Yellow solid (0.401 g, 90%), mp: 150–152 °C; ^1^H NMR (500 MHz,) δ 9.92 (s, 1H, CH–N), 9.10 (s, 1H, Ar–H), 8.34 (dd, *j* = 2 and 9 Hz, 2H, Ar–H), 8.13 (t, *j* = 7.5 Hz, 3H, Ar–H), 8.03 (s, 1H, Ar–H), 7.93 (td, *j* = 1 and 12 Hz, 2H, Ar–H), 7.76 (dd, *j* = 2 and 9 Hz, 1H, Ar–H), 7.35 (dt, *j* = 2 and 15.5 Hz, 2H, Ar–H), 7.30 (dt, *j* = 1.5 and 13.5 Hz, 2H, Ar–H), 7.09 (t, *j* = 6 Hz, 1H, Ar–H), 5.79 (s, 2H, CH_2_), 2.34 (s, 3H, CH_3_). ^13^C NMR (126 MHz, DMSO) δ 13.50 (CH_3_), 42.55 (CH_2_), 112.24 (CH), 112.34 (Ar–C), 115.37 (Ar–C), 118.52 (Ar–C), 119.38 (C–N), 119.46 (Ar–C), 123.10 (Ar–C), 123.29 (Ar–C), 123.73 (Ar–C), 124.28 (Ar–C), 124.53 (Ar–C), 126.67 (Ar–C), 129.23 (Ar–C), 129.37 (C–N), 131.98 (Ar–C), 137.05 (Ar–C), 137.46 (Ar–C), 139.27 (Ar–C), 140.85 (CH), 151.50 (C–N), 163.23 (C=O). Anal. Calcd for C_28_H_21_N_7_O_3_: C, 66.79; H, 4.20; N, 19.47; O, 9.53%; Found: C, 67.06; H, 4.19; N, 19.55; O, 9.56%.

#### 5-Methyl-2-phenyl-4-((1-((1-(o-tolyl)-1*H*-1,2,3-triazol-4-yl)methyl)-1*H*-indol-3-yl)methylene)-2,4-dihydro-3*H*-pyrazol-3-one (**5h**)

Yellow solid (0.358 g, 83%), mp: 220–222 °C; ^1^H NMR (500 MHz,) δ 9.99 (s, 1H, CH–N), 8.94 (s, 1H, Ar–H), 8.23 (dd, *j* = 2.5 and 8.5 Hz, 1H, Ar–H), 8.12 (s, 1H, Ar–H), 8.04 (td, *j* = 1 and 12 Hz, 2H, Ar–H), 7.86 (dd, *j =* 2 and 9 Hz, 1H, Ar–H), 7.76 (d, *j* = 8 Hz and 1H, Ar–H), 7.44 (t, *j* = 8 Hz, 2H, Ar–H), 7.38 (dd, *j* = 3 and 8 Hz, 3H, Ar–H), 7.19 (t, *j* = 7.5 Hz, 1H, Ar–H), 5.83 (s, 2H, CH_2_), 2.43 (s, 3H, CH_3_), 2.37 (s, 3H, CH_3_). Anal. Calcd for C_29_H_24_N_6_O: C, 73.71; H, 5.12; N, 17.78; O, 3.39%; Found: C, 74.00; H, 5.13; N, 17.71; O, 3.40%.

#### 4-((1-((1-(2-Methoxyphenyl)-1*H*-1,2,3-triazol-4-yl)methyl)-1*H*-indol-3-yl)methylene)-5-methyl-2-phenyl-2,4-dihydro-3*H*-pyrazol-3-one (**5i**)

Yellow solid (0.375 g, 87%), mp: 230–232 °C; ^1^H NMR (500 MHz,) δ; 9.99 (s, 1H, CH–N), 9.92 (d, *j* = 5 Hz, 1H, Ar–H), 8.89 (s, 1H, Ar–H), 8.23 (dd, *j* = 2.5 and 8.5 Hz, 1H, Ar–H), 8.11 (dd, *j* = 2 and 11 Hz, 1H, Ar–H), 8.02 (dd, *j* = 2.5 and 11 Hz, 2H, Ar–H), 7.87 (dd, *j* = 2 and 9 Hz, 1H, Ar–H), 7.78 (dd, *j* = 2 and 9 Hz, 1H, Ar–H), 7.45 (t, *j* = 7 Hz, 2H, Ar–H), 7.40–7.36 (m, 2H, Ar–H), 7.19 (t, *j* = 7.5 Hz, 2H, Ar–H), 7.12 (dd, *j* = 2 and 9 Hz, 1H, Ar–H), 5.82 (s, 2H, CH_2_) ^13^C NMR (126 MHz, DMSO) δ 13.48 (CH3), 56.02 (CH3), 97.94 (CH2), 109.06 (CH), 112.17 (Ar–C), 115.34 (Ar–C), 118.59 (C–N), 122.38 (Ar–C), 124.57 (Ar–C), 129.26 (C–N), 137.11 (Ar–C), 139.26 (Ar–C), 143.18 (CH), 151.53 (C–N), 163.24 (C–O), 169.46 (C=O). Anal. Calcd for C_29_H_24_N_6_O_2_: C, 71.30; H, 4.95; N, 17.20; O, 6.55%; Found: C, 71.09; H, 4.96; N, 17.27; O, 6.57%.

#### 4-((1-((1-(3,4-Dimethylphenyl)-1*H*-1,2,3-triazol-4-yl)methyl)-1*H*-indol-3-yl)methylene)-5-methyl-2-phenyl-2,4-dihydro-3*H*-pyrazol-3-one (**5j**)

Yellow solid (0.361 g, 84%), mp: 208–210 °C; ^1^H NMR (500 MHz, DMSO) δ ^13^C NMR (126 MHz, DMSO) δ 13.46 (CH3), 42.24 (CH3), 77.64 (CH3), 78.15 (CH2), 112.04 (CH), 112.17 (CH), 112.20 (C–N), 112.36 (Ar–C), 112.76 (Ar–C), 118.54 (Ar–C), 118.57 (Ar–C), 118.61 (Ar–C), 119.32 (Ar–C), 119.38 (Ar–C), 119.48 (Ar–C), 119.58 (Ar–C), 123.13 (Ar–C), 123.22 (Ar–C), 124.25 (Ar–C), 124.31 (Ar–C), 124.54 (Ar–C), 124.59 (Ar–C), 126.92 (Ar–C), 128.75 (Ar–C), 129.24 (Ar–C), 129.98 (Ar–C), 131.44 (C–N), 132.10 (Ar–C), 134.36 (Ar–C), 136.78 (Ar–C), 136.96 (Ar–C), 139.25 (Ar–C), 139.95 (Ar–C), 140.68 (Ar–C), 142.63 (CH), 151.52 (C–N), 163.21 (C=O). Anal. Calcd for C_30_H_26_N_6_O: C, 74.05; H, 5.39; N, 17.27; O, 3.29%; Found: C, 74.20; H, 5.37; N, 17.34; O, 3.28%.

#### 4-((1-((1-Benzyl-1*H*-1,2,3-triazol-4-yl)methyl)-1*H*-indol-3-yl)methylene)-5-methyl-2-phenyl-2,4-dihydro-3*H*-pyrazol-3-one (**5k**)

Yellow solid (0.379 g, 90%), mp: 210–212 °C; ^1^H NMR (500 MHz, DMSO) δ 9.90 (s, 1H, CH–N), 8.31 (s, 1H, Ar–H), 8.22–8.20 (m, 1H, Ar–H), 8.09 (s, 1H, Ar–H), 8.02 (dd, *j* = 1.5 and 10 Hz, 2H, Ar–H), 7.83–7.79 (m, 1H, Ar–H), 7.44 (dt, *j* = 2 and 16 Hz, 2H, Ar–H), 7.38–7.28 (m, 6H, Ar–H), 7.19 (tt, *j* = 1.5 and 17.5 Hz, 2H, Ar–H), 5.73 (s, 2H, CH_2_), 5.59 (s, 2H, CH_2_), 2.42 (s, 3H, CH_3_). ^13^C NMR (126 MHz, DMSO) δ 13.48 (CH3), 42.55 (CH2), 53.34 (CH2), 112.12 (CH), 112.31 (C–N), 118.59 (Ar–C), 119.19 (Ar–C), 119.40 (Ar–C), 123.08 (Ar–C), 124.19 (Ar–C), 124.56 (Ar–C), 124.65 (Ar–C), 128.36 (Ar–C), 128.63 (Ar–C), 129.23 (Ar–C), 129.25 (Ar–C), 129.30 (C–N), 136.31 (Ar–C), 136.73 (Ar–C), 137.04 (Ar–C), 139.27 (Ar–C), 140.69 (CH), 151.50 (C–N), 163.22 (C=O) Anal. Calcd for C_29_H_24_N_6_O: C, 73.71; H, 5.12; N, 17.78; O, 3.39%; Found: C, 73.42; H, 5.10; N, 17.74; O, 3.40%.

#### 4-((3-((3-Methyl-5-oxo-1-phenyl-1,5-dihydro-4*H*-pyrazol-4-ylidene)methyl)-1*H*-indol-1-yl)methyl)-1*H*-1,2,3-triazol-1-yl butyrate (**5l**)

Yellow solid (0.438 g, 93%), mp: 200–210 °C; ^1^H NMR (500 MHz, DMSO) δ 9.96 (s, 1H, CH–N), 8.25 (s, 1H, Ar–H), 8.23–8.21 (m, 1H, Ar–H), 8.11 (s, 1H, Ar–H), 8.03 (td, *j* = 2.5 and 10 Hz, 2H, Ar–H), 7.83 (dt, *j* = 2.5 Hz and 9.5 Hz, 1H, Ar–H), 7.45 (dt, *j* = Hz and 11.5 Hz, 2H, Ar–H), 7.38 (dt, *j* = 2 Hz and 11.5 Hz, 2H, Ar–H), 7.19 (tt, *j* = 1.5 Hz and 11.5 Hz, 2H, Ar–H), 5.79 (s, 2H, CH_2_), 5.38 (s, 2H, CH_2_), 4.15 (q, *j* = 7.5 Hz, 2h, CH_2_), 2.43 (s, 3H, CH_3_), 1.18 (t, *j* = 7 Hz, 3H, CH_3_). ^13^C NMR (126 MHz, DMSO) δ 13.50 (CH3), 14.37 (CH3), 42.50 (CH2), 50.90 (CH2), 61.95 (CH2), 112.16 (CH), 112.37 (C–N), 118.55 (Ar–C), 119.26 (Ar–C), 119.42 (Ar–C), 123.05 (Ar–C), 124.18 (Ar–C), 124.52 (Ar–C), 125.76 (Ar–C), 129.25 (Ar–C), 129.33 (Ar–C), 136.73 (Ar–C), 137.04 (Ar–C), 139.30 (Ar–C), 140.79 (Ar–C), 142.63 (CH), 151.49 (C–N), 163.24 (C=O), 167.58 (C=O). Anal. Calcd for C_29_H_24_N_6_O_2_: C, 71.30; H, 4.95; N, 17.20; O, 6.55, %; Found: C, 71.09; H, 4.94; N, 17.18; O, 6.58%

#### 5-Methyl-2-phenyl-4-((1-((1-(2,4,5-trifluorophenyl)-1*H*-1,2,3-triazol-4-yl)methyl)-1*H*-indol-3-yl)methylene)-2,4-dihydro-3*H*-pyrazol-3-one (**5m**)

Yellow solid (0.380 g, 91%), mp: 200–202 °C; ^1^H NMR (500 MHz, DMSO) δ 9.97 (s, 1H, CH–N), 9.92 (s, 1H, Ar–H), 8.80 (s, 1H, Ar–H), 8.24 (t, *j* = 6.5 Hz, 1H, Ar–H), 8.17 (s, 1H, Ar–H), 8.12 (d, *j* = 3 Hz, 1H, Ar–H), 8.02 (d, *j* = 8.5 Hz, 2H, Ar–H), 7.90 (d, *j =* 8 Hz, 1H, Ar–H), 7.74 (d, *j* = 7.5 Hz, 1H, Ar–H), 7.47–7.38 (m, 3H, Ar–H), 7.19 (t, *j* = 7.5 Hz, 1H, Ar–H), 5.88 (s, 1H, −CH−), 5.42 (s, 1H,-CH−), 2.43 (s, 3H, CH_3_). ^13^C NMR (126 MHz, DMSO) δ 13.50 (CH3), 42.32 (CH2), 112.21 (CH), 112.37 (C–N), 113.45 (Ar–C), 118.54 (Ar–C), 118.56 (Ar–C), 119.27 (Ar–C), 119.43 (Ar–C), 123.17 (Ar–C), 123.75 (Ar–C), 124.25 (Ar–C), 124.47 (Ar–C), 124.54 (Ar–C), 125.78 (Ar–C), 127.13 (Ar–C), 129.26 (Ar–C), 129.36 (C–N), 135.41 (Ar–C), 135.48 (Ar–C), 136.93 (Ar–C), 137.02 (Ar–C), 139.25 (Ar–C), 140.41 (Ar–C), 142.48 (CH), 151.51 (C–N), 163.19 (C=O). Anal. Calcd for C_28_H_19_F_3_N_6_O: C, 65.62; H, 3.74; N, 16.40; O, 3.12%; Found: C, 65.42; H, 3.75; N, 16.37; O, 3.11%

#### 5-Methyl-2-phenyl-4-((1-((1-(2,4,6-tribromophenyl)-1*H*-1,2,3-triazol-4-yl)methyl)-1*H*-indol-3-yl)methylene)-2,4-dihydro-3*H*-pyrazol-3-one (**5n**)

Yellow solid (0.366 g, 89%), mp: 200–202 °C; ^1^H NMR (500 MHz,) δ 9.88 (s, 1H, CH–N), 8.71 (s, 1H, Ar–H), 8.27 (s, 1H, Ar–H), 8.24 (dd, *j* = 2 and 9 Hz, 1H, Ar–H), 8.12 (s, 1H, Ar–H), 7.90 (dd, *j* = 5 and 14 Hz, 2H, Ar–H), 7.85 (dd, *j* = 1.5 and 9 Hz, 1H, Ar–H), 7.46–7.39 (m, 5H, Ar–H), 7.18 (t, *j* = 7.5 Hz, 1H, Ar–H), 5.88 (s, 2H, CH_2_), 2.43 (s, 3H, CH_3_). ^13^C NMR (126 MHz, DMSO) δ 13.49 (CH3), 18.44, 42.40 (CH2), 112.19 (CH), 112.35 (C–N), 118.49, 119.26, 119.45, 121.54, 123.14, 124.25, 124.51, 124.85, 126.68, 129.22, 129.35 (C–N), 133.29 (Ar–C), 136.79 (Ar–C), 136.87 (Ar–C), 137.06 (Ar–C), 139.28 (Ar–C), 140.65 (Ar–C), 141.88 (Ar–C), 146.54 (CH), 151.50 (C–N), 163.23 (C=O). Anal. Calcd for C_28_H_19_Br_3_N_6_O: C, 48.37; H, 2.75; N, 12.09; O, 2.30%; Found: C, 48.18; H, 2.47; N, 12.07; O, 2.29; S, 7.04%

#### 5-Methyl-4-((1-((1-(2-methyl-5-nitrophenyl)-1*H*-1,2,3-triazol-4-yl)methyl)-1*H*-indol-3-yl)methylene)-2-phenyl-2,4-dihydro-3*H*-pyrazol-3-one (**5o**)

Yellow solid (0.405 g, 89%), mp: 210–212 °C; ^1^H NMR (500 MHz,) δ 9.45 (s, 1H, CH–N), 8.85 (s, 1H, Ar–H), 8.35 (dd, *j* = 2.5 and 12 Hz, 2H, Ar–H), 8.24 (dd, *j* = 2 and 8 Hz, 1H, Ar–H), 8.13 (s, 1H, Ar–H), 8.01 (d, *j* = 8.5 Hz, 2H, Ar–H), 7.90 (dd, *j* = 1.5 and 9 Hz, 1H, Ar–H), 7.80 (d, *j* = 8.5 Hz, 1H, Ar–H), 7.46–7.38 (m, 3H, Ar–H), 7.18 (t, *j* = 7 Hz, 2H, Ar–H), 5.87 (s, 2H, CH_2_), 2.43 (s, 3H, CH_3_), 2.32 (s, 3H, CH_3_). ^13^C NMR (126 MHz, DMSO) δ 13.49 (CH3), 18.44 (CH3), 42.40 (CH2), 112.19 (CH), 112.35 (C–N), 118.49 (Ar–C), 119.26 (Ar–C), 119.45 (Ar–C), 121.54 (Ar–C), 123.14 (Ar–C), 124.25 (Ar–C), 124.51 (Ar–C), 124.85 (Ar–C), 126.68 (Ar–C), 129.22 (Ar–C), 129.35 (C–N), 133.29 (Ar–C), 136.79 (Ar–C), 136.87 (Ar–C), 137.06 (Ar–C), 139.28 (Ar–C), 140.65 (Ar–C), 141.88 (Ar–C), 146.54 (CH), 151.50 (C–N), 163.23 (C=O). Anal. Calcd for C_29_H_23_N_7_O_3_: C, 67.30; H, 4.48; N, 18.94; O, 9.27%; Found: C, 67.10; H, 4.47; N, 18.86; O, 9.31%

### Biological Screening

#### *In Vitro* Antimicrobial Screening

All the synthesized compounds were screened against Gram-positive bacteria, Gram-negative bacteria, and fungi by the use of the broth dilution method. Mueller–Hinton broth was used as the nutrient medium to grow and dilute the compound suspension for the test bacteria. Serial dilutions were prepared in primary and secondary screening. The compound was run in the primary screening at three different concentrations: 1000, 500, 250, and 125 μg mL^–1^. Further, the compounds that were found to be active in primary screening were similarly diluted to obtain 100, 62.5, 50, 25, and 10 μg mL^–1^ concentrations for secondary screening. DMSO was used as a diluent to get the desired concentration of drugs to test upon standard bacterial strains. The experiment was repeated three times. The turbidity of each sample-containing tube is measured in comparison with the negative control tube. The minimum concentration of the compound that can inhibit the growth of the microorganism completely (99%) was considered and recorded as the minimum inhibitory concentration (MIC, μg mL^–1^), which was measured visibly. All required strains were purchased from MTCC (Microbial Type Culture Collection), Institute of Microbial Technology, Chandigarh.^40^

#### *In Vitro* Bactericidal Activity

A pure culture of a specified microorganism is grown overnight and then diluted in growth-supporting broth (typically Mueller Hinton Broth) to a concentration between 1 × 10^5^ and 1 × 10^6^ cfu/mL. Further 1:1 dilutions are made in test tubes or 96-well microtiter plates. All dilutions of the test product(s) are inoculated with equal volumes of the specified microorganism. A positive and negative control tube or well is included for every test microorganism to demonstrate adequate microbial growth over the course of the incubation period and media sterility, respectively. An aliquot of the positive control is plated and used to establish a baseline concentration of the microorganism used. The tubes or microtiter plates are then incubated at the appropriate temperature and duration. Turbidity indicates growth of the microorganism, and the MIC is the lowest concentration where no growth is visually observed. To determine the MBC, the dilution representing the MIC and at least two of the more concentrated test product dilutions are plated and enumerated to determine the viable CFU/mL. The MBC is the lowest concentration that demonstrates a predetermined reduction (such as 99.9%) in CFU/mL when compared to the MIC dilution.

#### *In Vitro* Cytotoxicity Measurement of Compounds Using Hoechst and Propidium Iodide

##### Cell Lines and Supplements

All of the chemical reagents were commercially purchased from the following sources. The human neuroblastoma cell line (SH-SY5Y) was purchased from ATCC, USA. Fetal bovine serum (FBS), penicillin, streptomycin, trypsin (0.25%), and DMSO (cell culture grade) were obtained from Sigma-Aldrich. Propidium iodide (PI) Hoechst and trypsin-EDTA 0.25% were purchased from Invitrogen, USA. The culture media DMEM/F-12 (Dulbecco’s Modified Eagle Medium/Nutrient Mixture F-12) were purchased from Corning, USA. Penicillin and streptomycin (antibiotics) used in this study were purchased from Gibco.

#### Cell Culture

SH-SY5Y cells were grown and maintained in T-75 flasks in a cell culture incubator at 37 °C under 5% carbon dioxide (CO_2_). The cell culture medium used was DMEM/F-12 supplemented with 10% (v/v) fetal bovine serum (FBS) and 1% penicillin/streptomycin (v/v). Trypsin-EDTA (0.25%) was used for harvesting the cultured cells. For cell culture experiments, 96-well plates were used.

#### *In Vitro* Cytotoxicity Measurement of Compounds and Confocal Imaging of SH-SY5Y Cells

For cytotoxicity measurements, SH-SY5Y cells were counted using a hemocytometer and cells were seeded in 96-well plates with 10,000 cells/well.^47,48^ Cells were left for incubation for 12 h at 37 °C under 5% carbon dioxide (CO_2_) for attachment to the bottom of the plates. Thereafter, cells were treated on compounds **5a**–**o** as a treatment group, with untreated cells as the negative control, DMSO as the vehicle, and hydrogen peroxide (10 mM) as a positive control. Cells were treated with **100 μg/mL** of all compounds and left to incubate for 24 h.^49^ Thereafter, cells were stained with Hoechst/PI (1 μg/mL of their final concentration) 10 μL/well and left for 1 h of incubation. This method of cytotoxicity measurement does not require washing steps, so after completion of incubation, the cytotoxicity measurements were performed using a multiplate reader (IN-Cell Analyzer 2000 Bioimager, v4.0 software, GE Healthcare). The images were in live-cell mode with a reader equipped with a 10× objective using two preselected excitation/emission filter sets: 380/535 nm for Hoechst and 555/645 nm for PI. Four adjacent fields were selected per well and a fluorescence channel with a 2 × 2 montage. Image acquisition and data processing were performed using an IN-Cell analyzer workstation with v3.7.2 software (GE Healthcare).^50^ This allowed for the segmentation of the images, providing the region of interest and the cytotoxicity percentages of cell death for each well. The experiment was replicated three times. The data obtained provide the % cytotoxicity of SH-SY5Y cells as a function of treatment. All of the experiments were performed in triplicates.

Confocal microscopy was further performed to validate the cytotoxicity results, In a separate experiment, 10,000 cells were seeded in each well in a 96-well plate and incubated, at 37 °C and 5% CO_2_ for 12 h for cells to adhere to the bottom of the plate. After completion of incubation, cells were treated with DMSO, H_2_O_2_ (10 mM), and compounds (**5a**–**o**). Cells were incubated for another 24 h similar to the cytotoxicity experiment. After 24 h, cells were stained with 10 μL/well of Hoechst/PI mixture (1 μg/mL of their final concentration), followed by 1 h of incubation. Confocal images were captured in 10× magnification using an LSM-700 Zeiss confocal microscope at Border Biomedical Research Center, University of Texas at El Paso.

## Supporting Information Available

Detailed characterization data for the synthesized derivatives, including ^1^H NMR and ^13^C NMR spectra, as well as HPLC data (PDF)
