# Supplementary material for: Antimicrobial Efficacy of 1,2,3-Triazole-Incorporated Indole-Pyrazolone against Drug-Resistant ESKAPE Pathogens: Design and Synthesis
Source: ACS Bio Med Chem Au. 2025 Jan 28;5(1):66–77. doi: 10.1021/acsbiomedchemau.4c00060 (PMC11843341; doi:10.1021/acsbiomedchemau.4c00060)
Supplement: Supplementary file 1 — bg4c00060_si_001.pdf [file bg4c00060_si_001.pdf]

Antimicrobial efficacy of 1,2,3-triazole incorporated indole-pyrazolone against drug-resistant ESKAPE pathogens: Design and synthesis

Dipti B. Upadhyay[a], Jaydeep A. Mokariya[a], Paras J. Patel[a], Subham G. Patel[a], Mehul P. Parmar[a], Disha P. Vala[a], Febe Ferro[b], Dhanji P. Rajani[c], Mahesh Narayan[d], Jyotish Kumar[d], Sourav Banerjee[b,*], Hitendra M. Patel [a,*]

[a]Department of Chemistry, Sardar Patel University, Vallabh Vidyanagar 388120, Gujarat, India.
[b]Division of Cancer Research, School of Medicine, University of Dundee, Dundee DD1 9SY, UK,
[c]Microcare Laboratory and Tuberculosis Diagnosis & Research Center, Surat, 395003, India
[d]Department of Chemistry and Biochemistry, The University of Texas at El Paso El Paso, TX 79968 USA
* Corresponding Authors: Hitendra M. Patel, E-mail: hm_patel@spuvvn.edu
Sourav Banerjee, Email: s.y.banerjee@dundee.ac.uk

Table of Contents

Sr. No.	Content	Page No.
1	^{1}H-NMR and ^{13}C{^{1}H}-NMR spectra of compound **5(a-o)**	1-16
2.	HPLC data of Compound **5(a-o)**	17-22

Copies of ^{1}H NMR and ^{13}C{^{1}H}- NMR spectra for compound 5a-5o

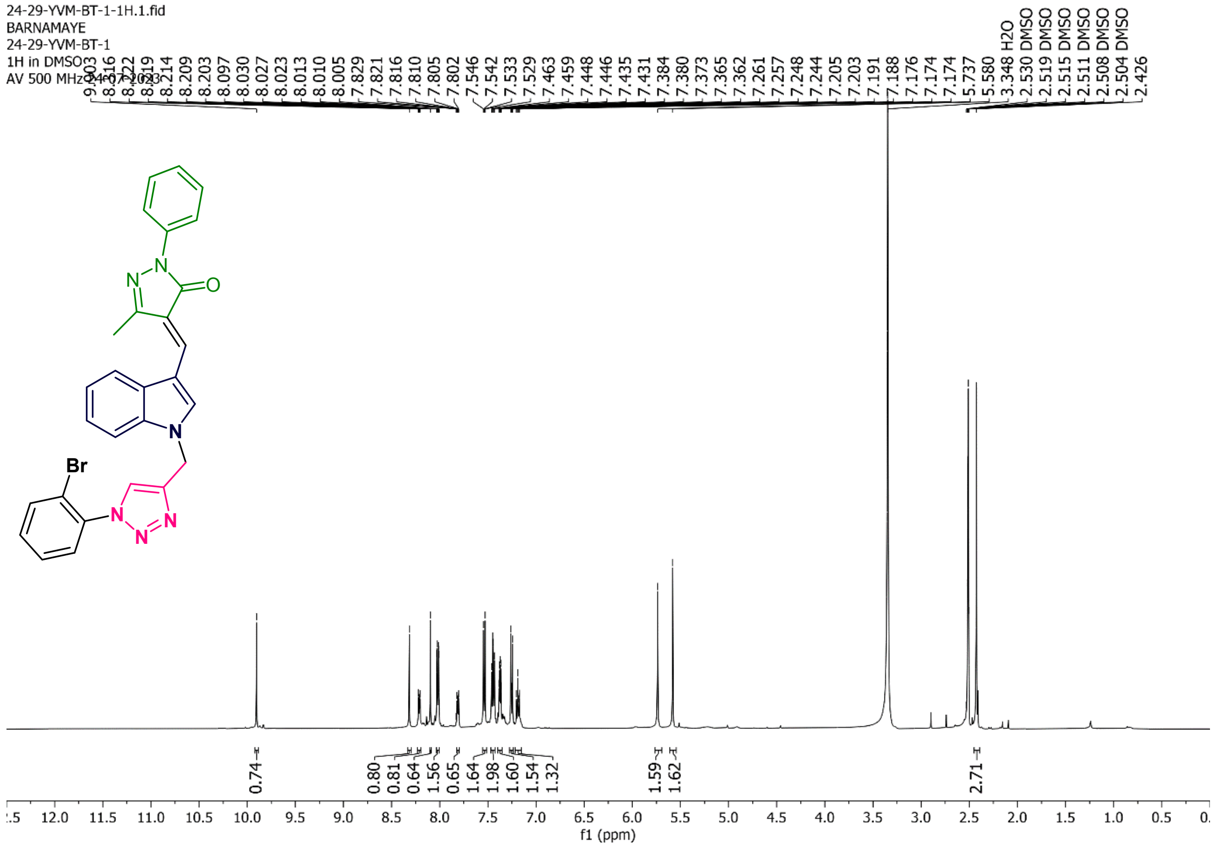

Figure 1. ^{1}H NMR spectrum of compound **5a** at 500 MHz in DMSO-d_6

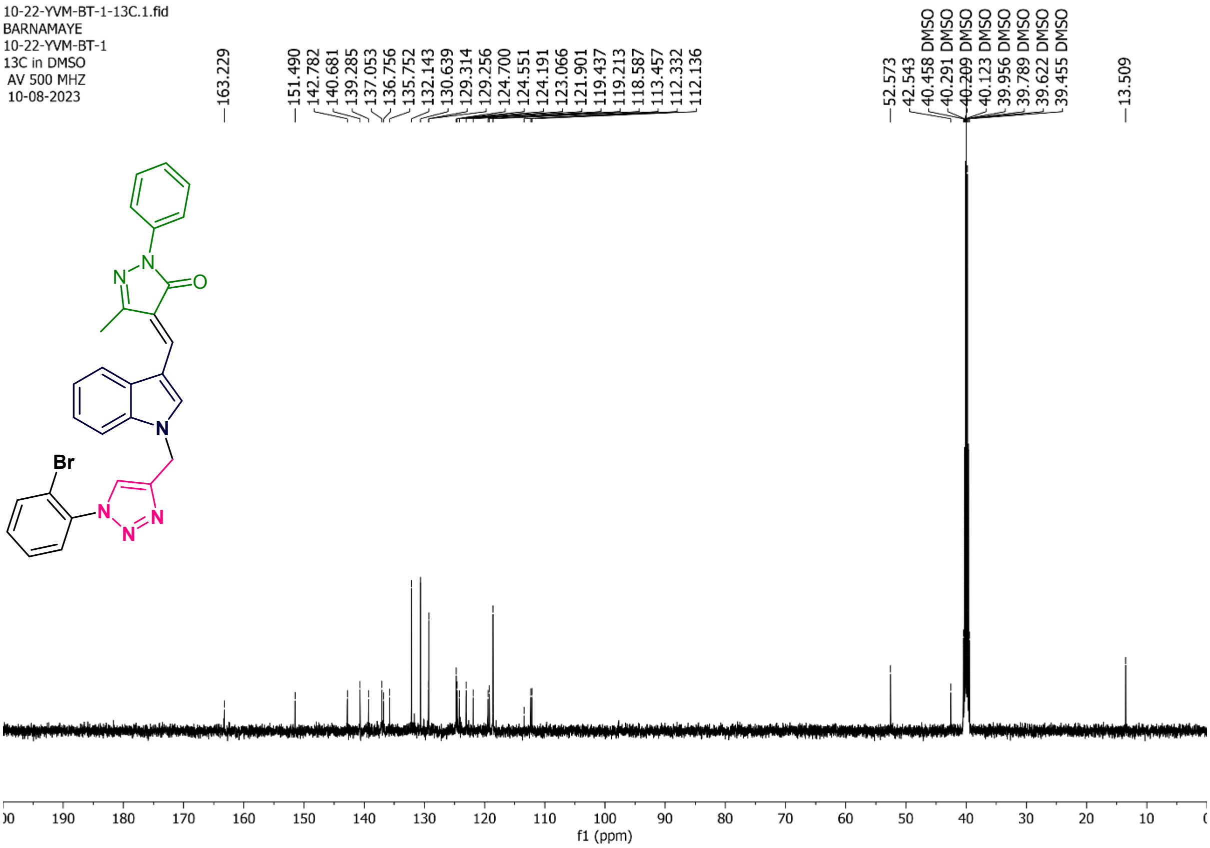

Figure 2. ^{13}C NMR spectrum of compound **5a** at 126 MHz in DMSO-d$_6$

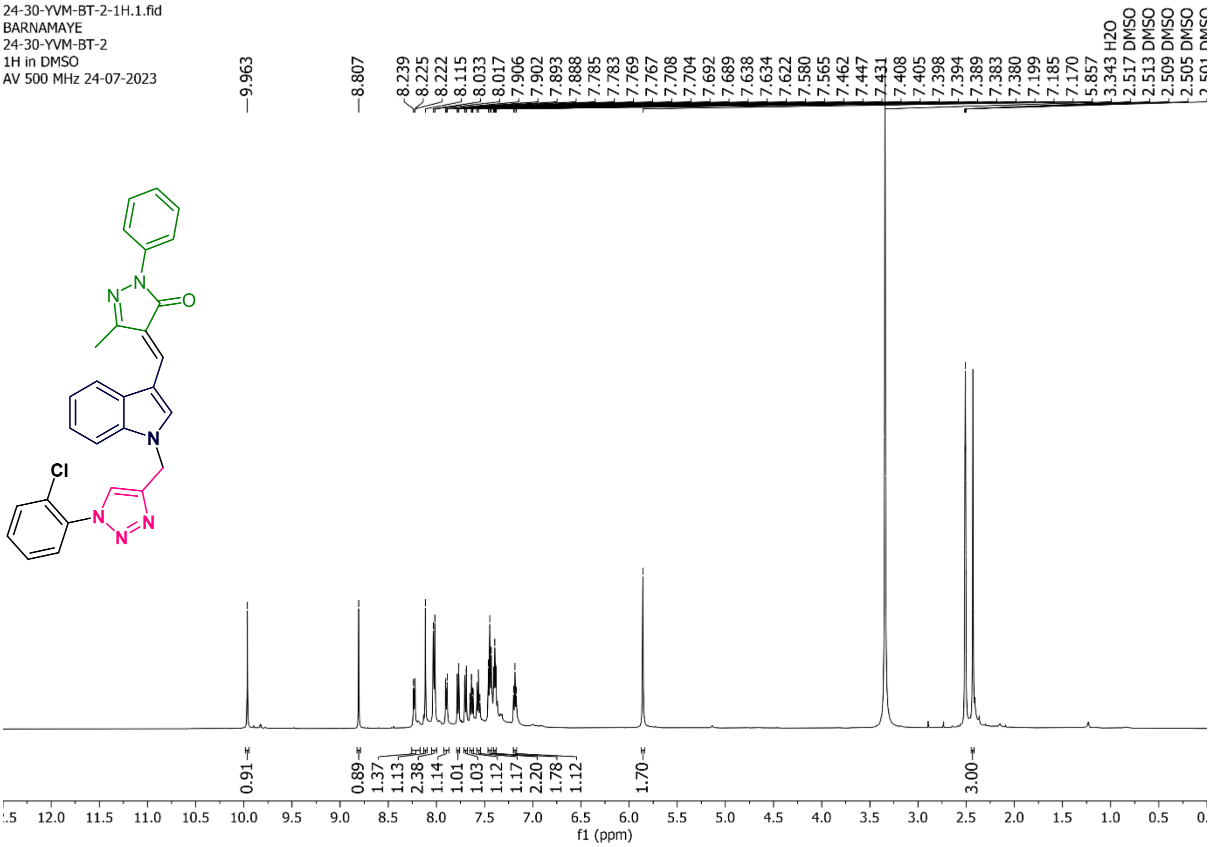

Figure 3. ^{1}H NMR spectrum of compound **5b** at 500 MHz in DMSO-d$_6$

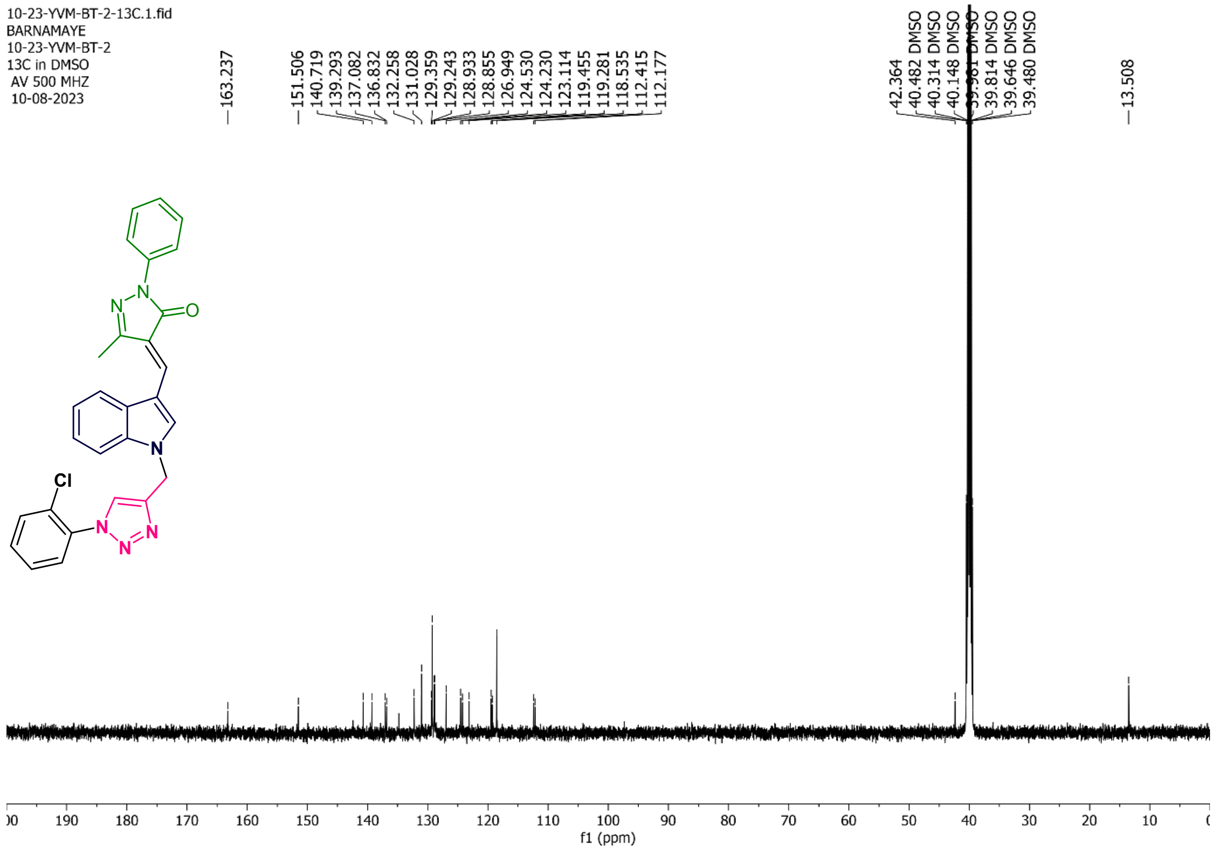

Figure 4. ^{13}C NMR spectrum of compound **5b** at 126 MHz in DMSO-d6

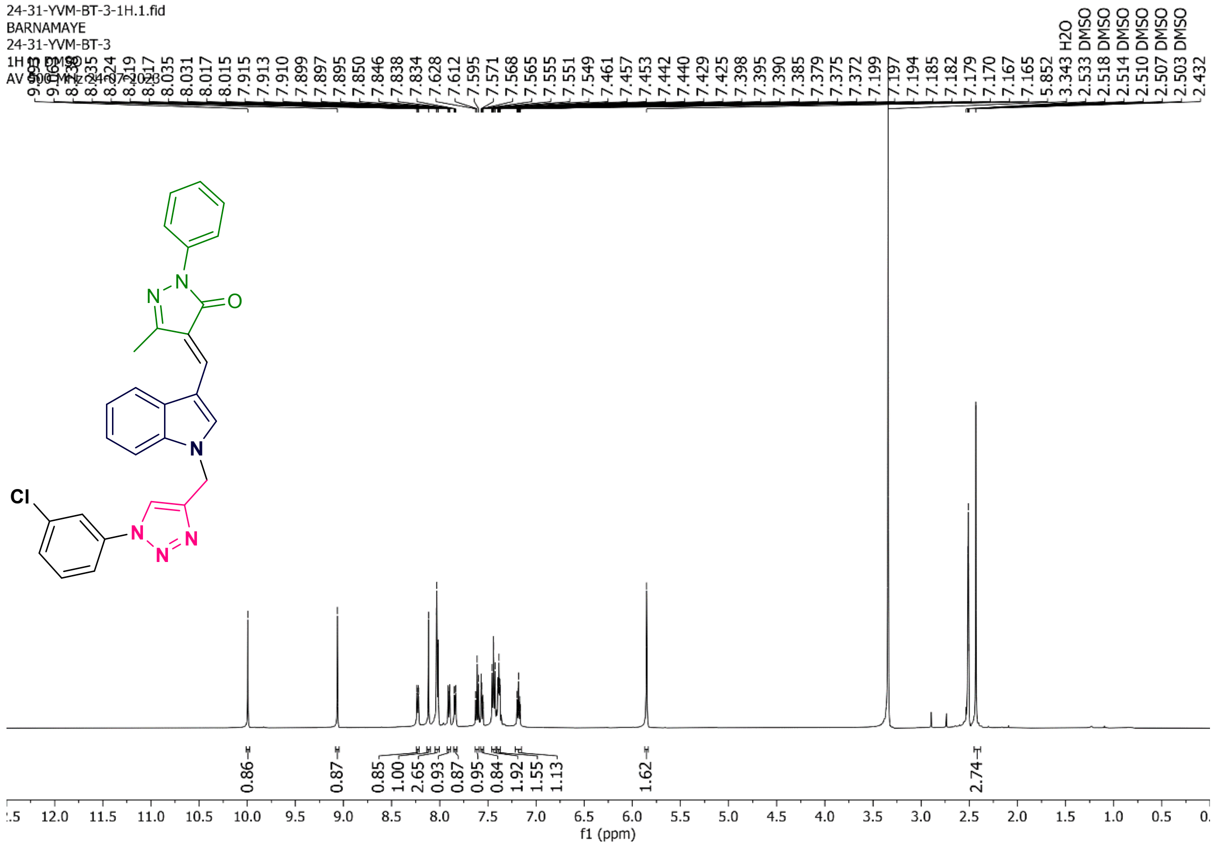

Figure 5. ^{1}H NMR spectrum of compound **5c** at 500 MHz in DMSO-d6

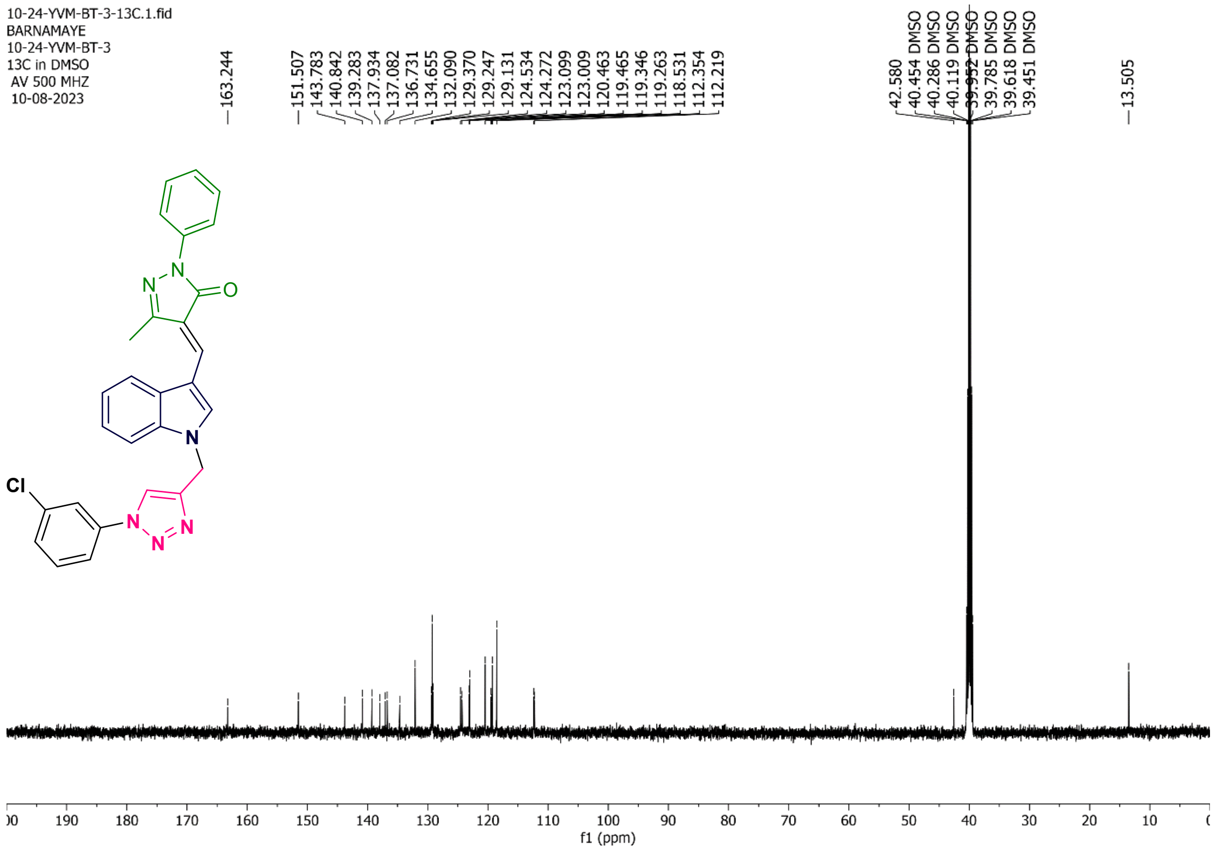

Figure 6. ^{13}C NMR spectrum of compound **5c** at 126 MHz in DMSO-d_6

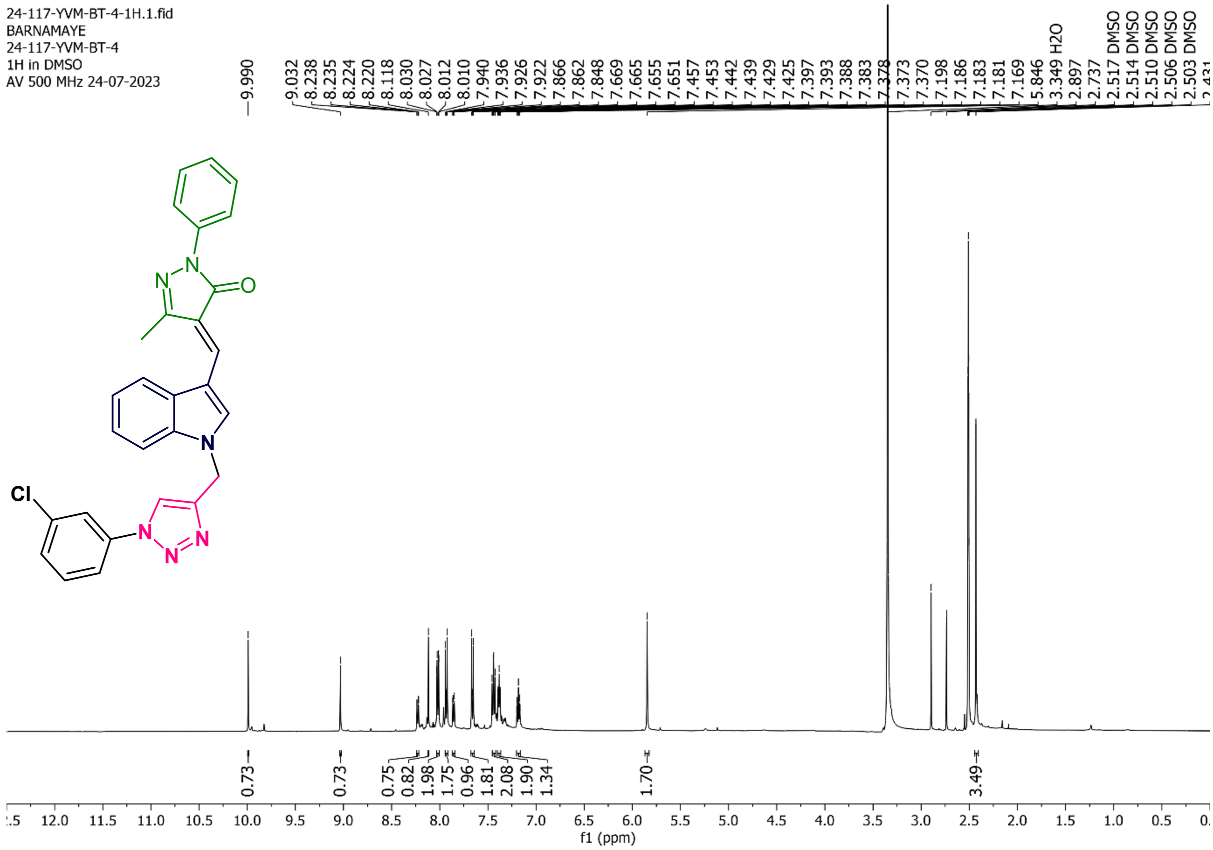

Figure 7. ^{1}H NMR spectrum of compound **5d** at 500 MHz in DMSO-d_6

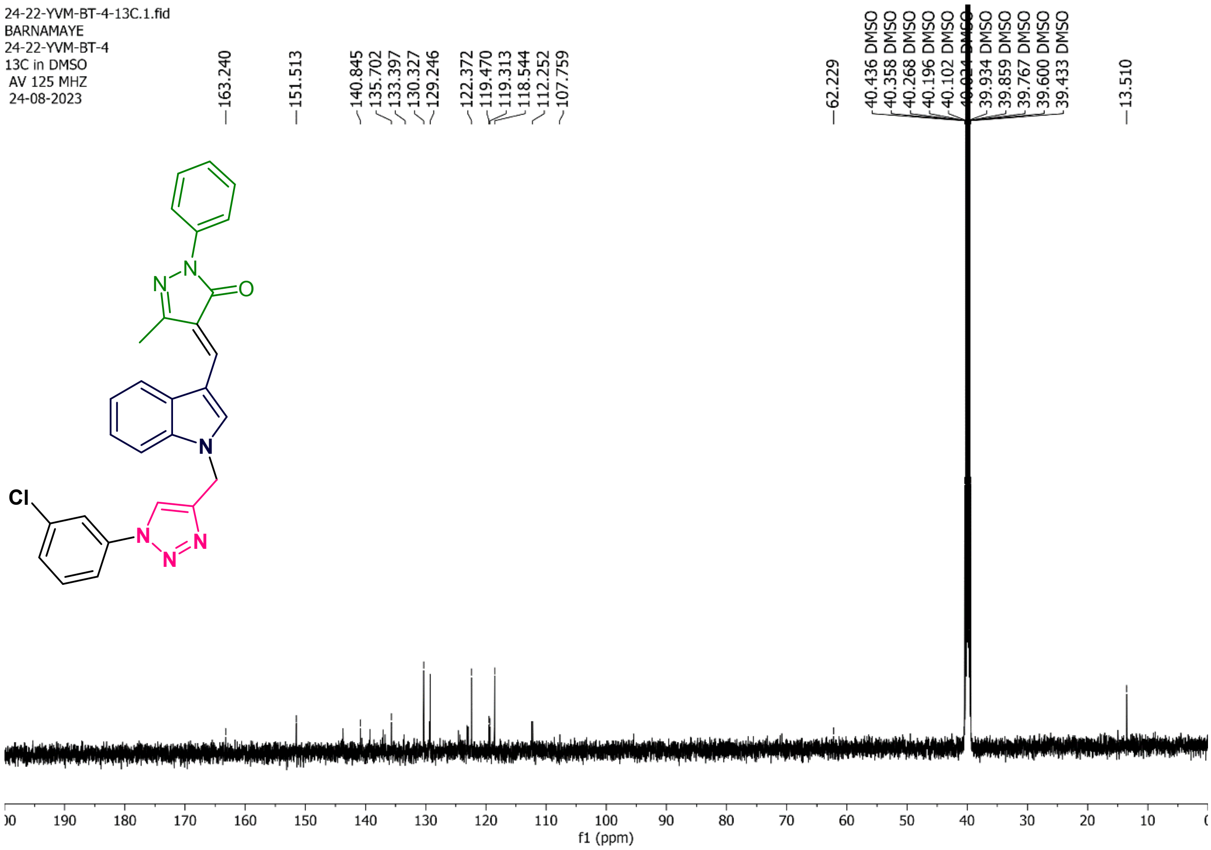

Figure 8. ^{13}C NMR spectrum of compound **5d** at 126 MHz in DMSO-d_6

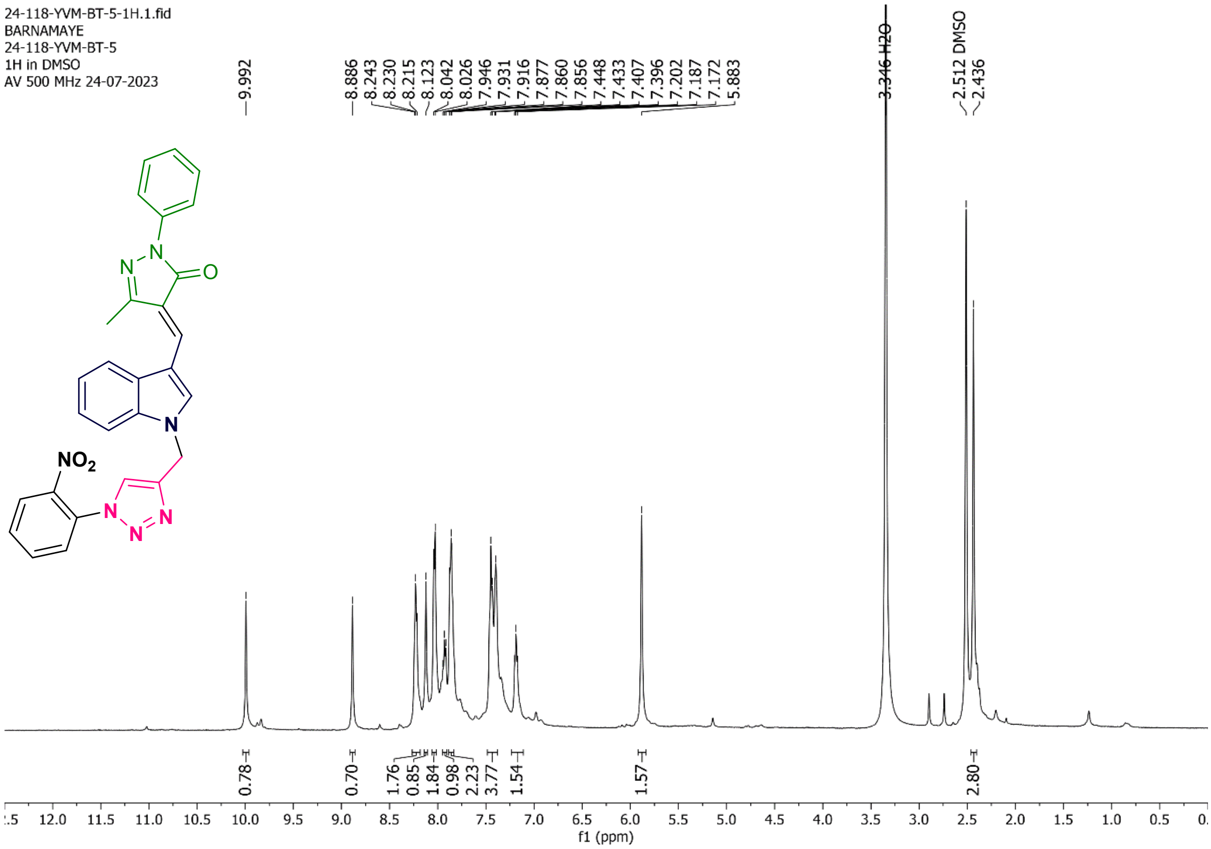

Figure 9. ^{1}H NMR spectrum of compound **5e** at 500 MHz in DMSO-d_6

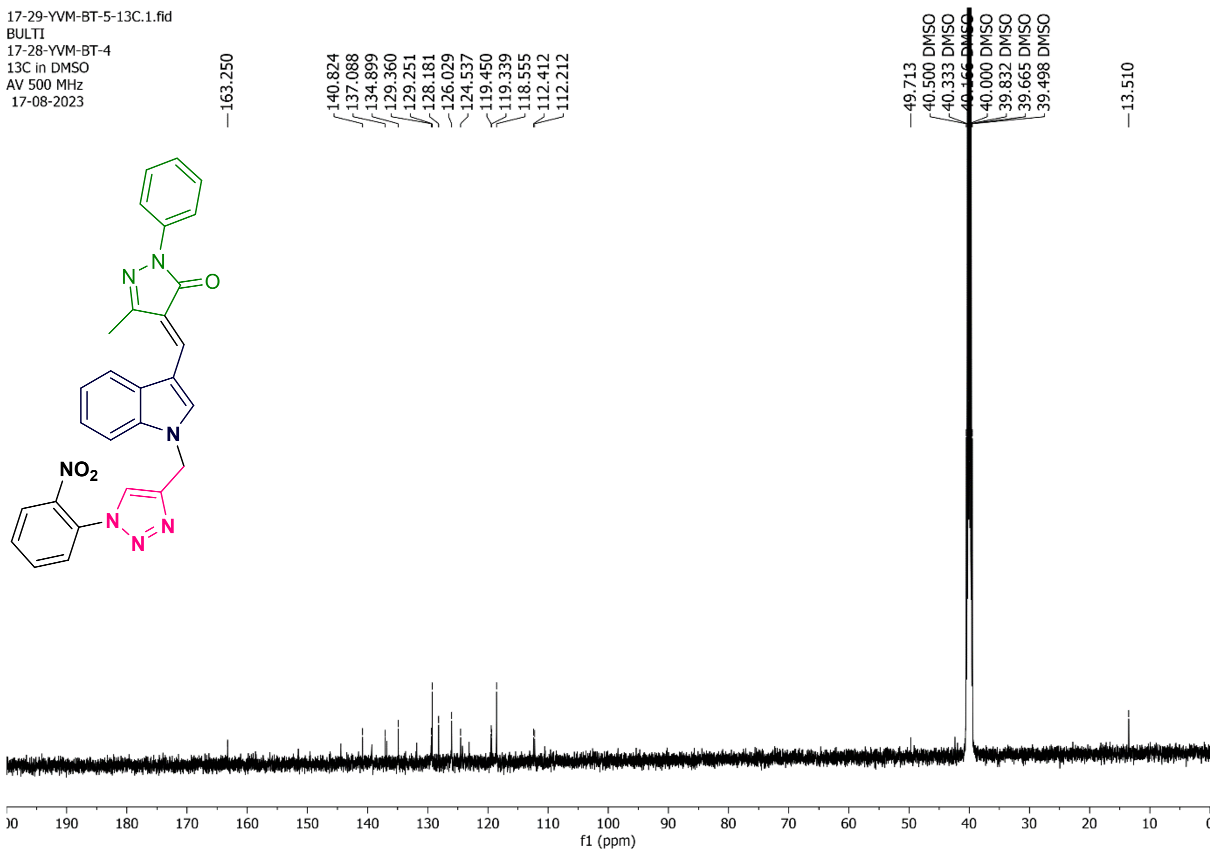

Figure 10. ^{13}C NMR spectrum of compound **5e** at 126 MHz in DMSO-d6

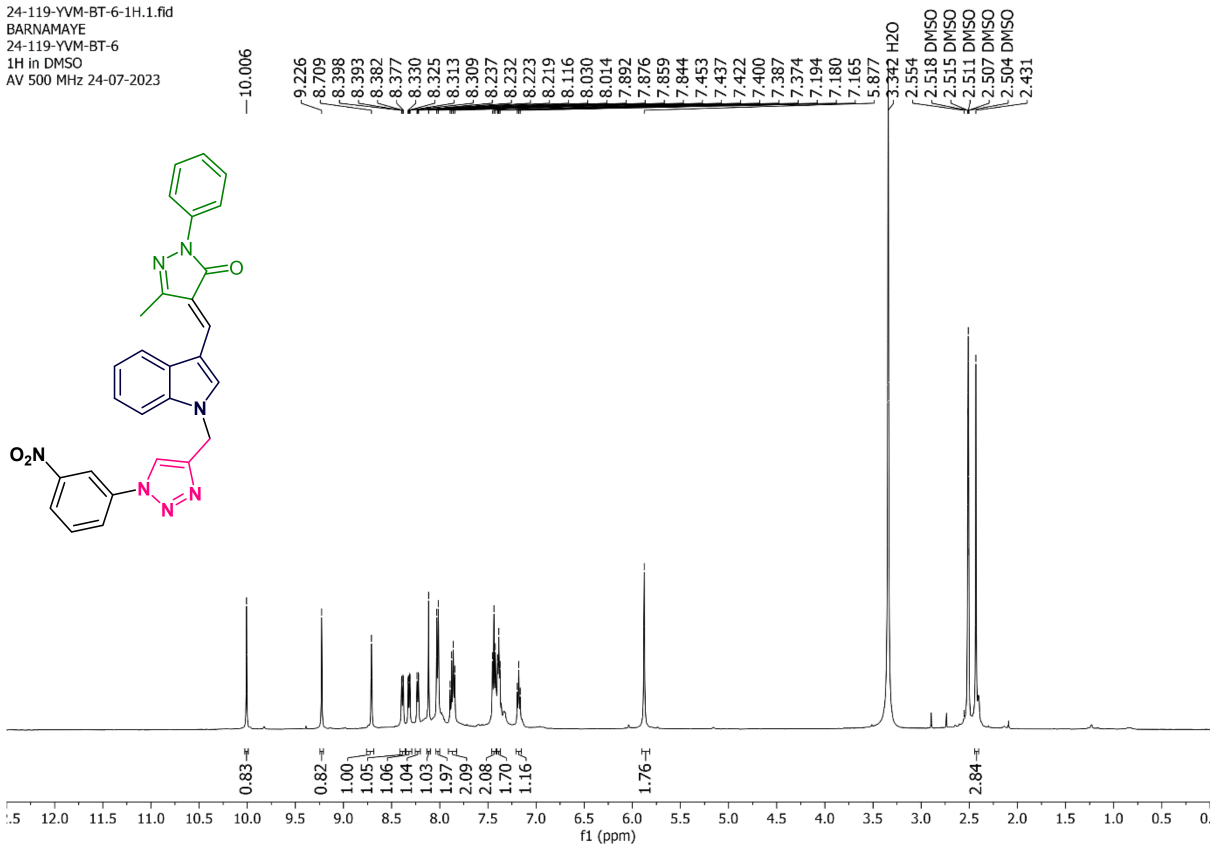

Figure 11. ^{1}H NMR spectrum of compound **5f** at 500 MHz in DMSO-d6

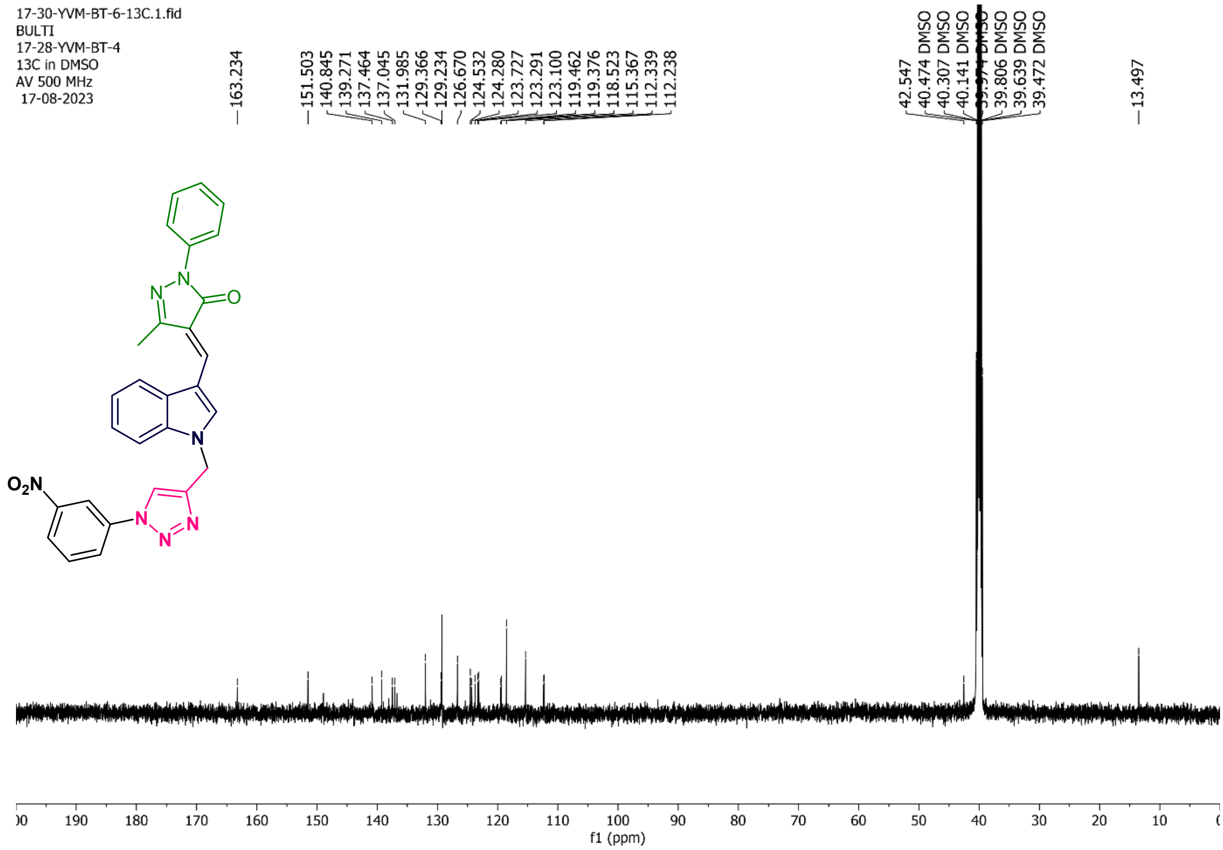

Figure 12. ^{13}C NMR spectrum of compound **5f** at 126 MHz in DMSO-d6

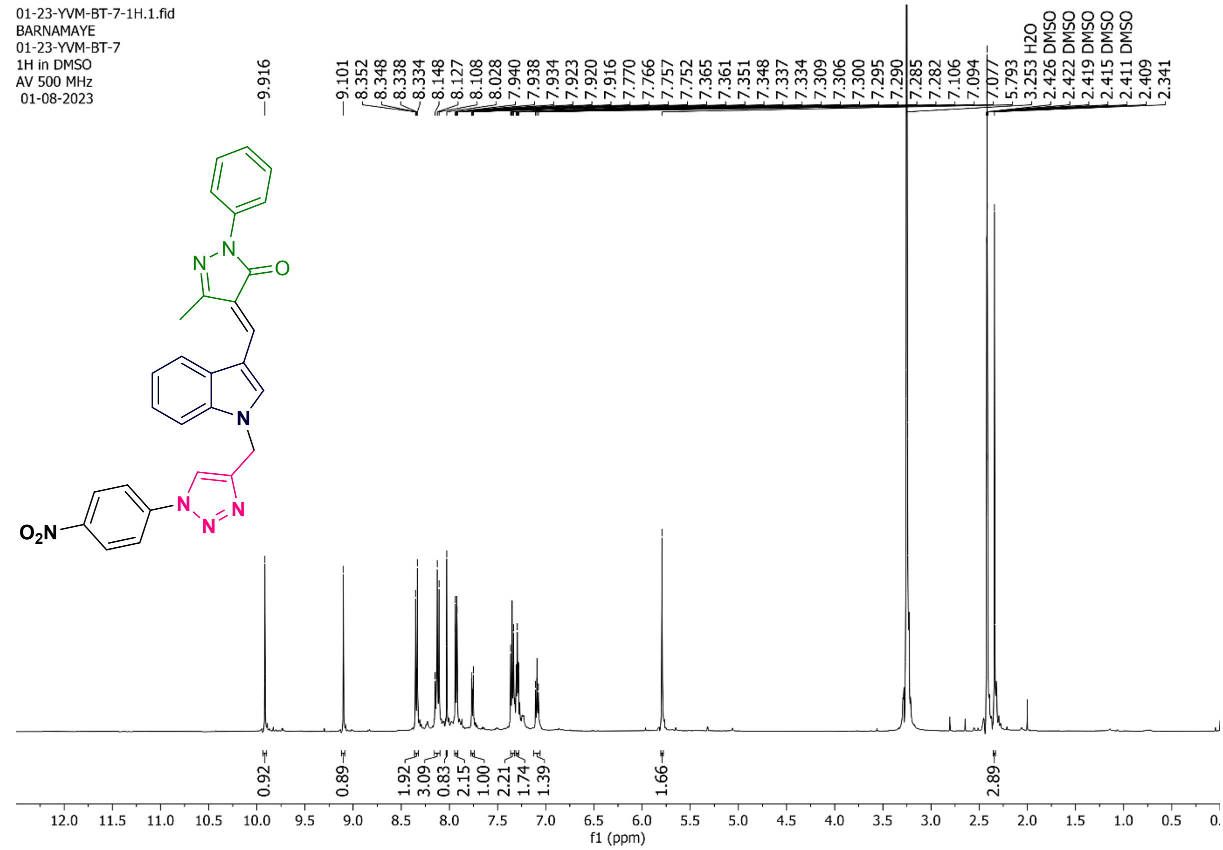

Figure 13. ^{1}H NMR spectrum of compound **5g** at 500 MHz in DMSO-d6

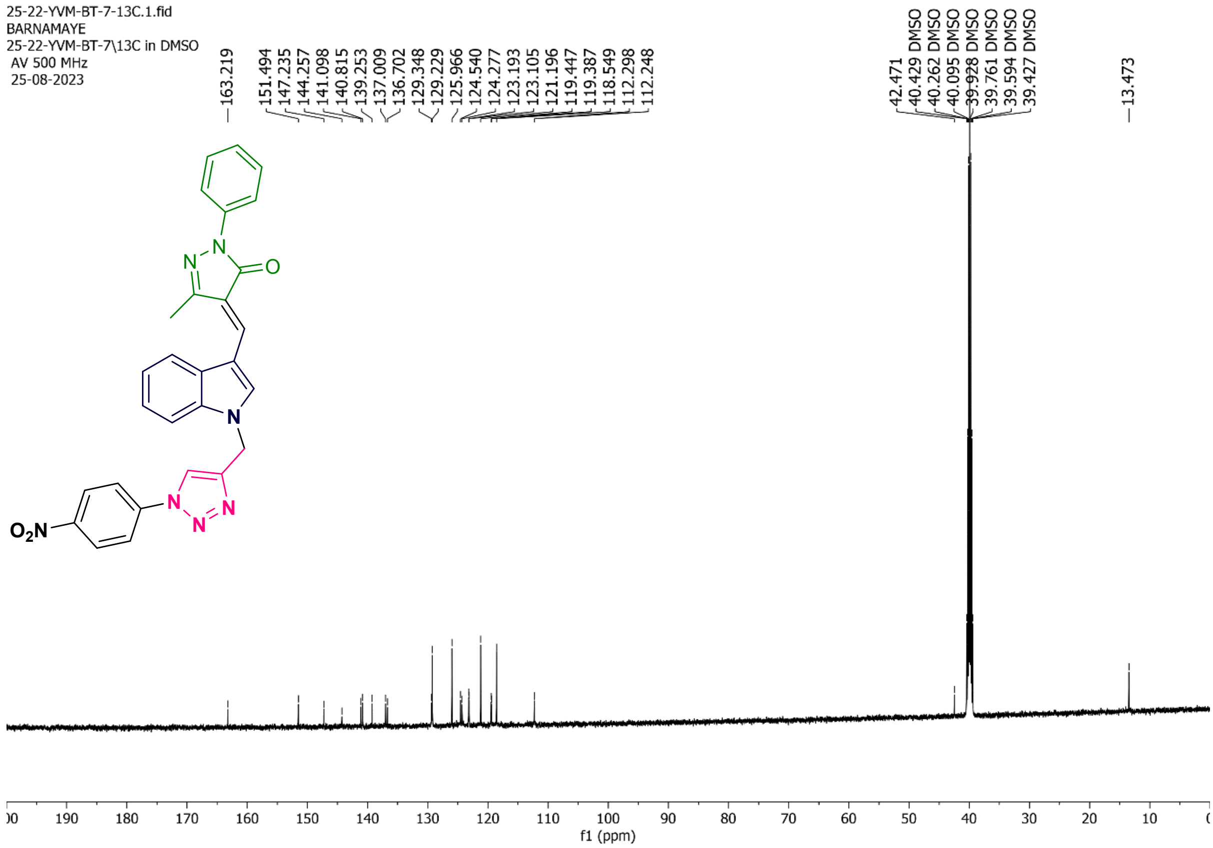

Figure 14. ^{13}C NMR spectrum of compound **5g** at 126 MHz in DMSO-d6

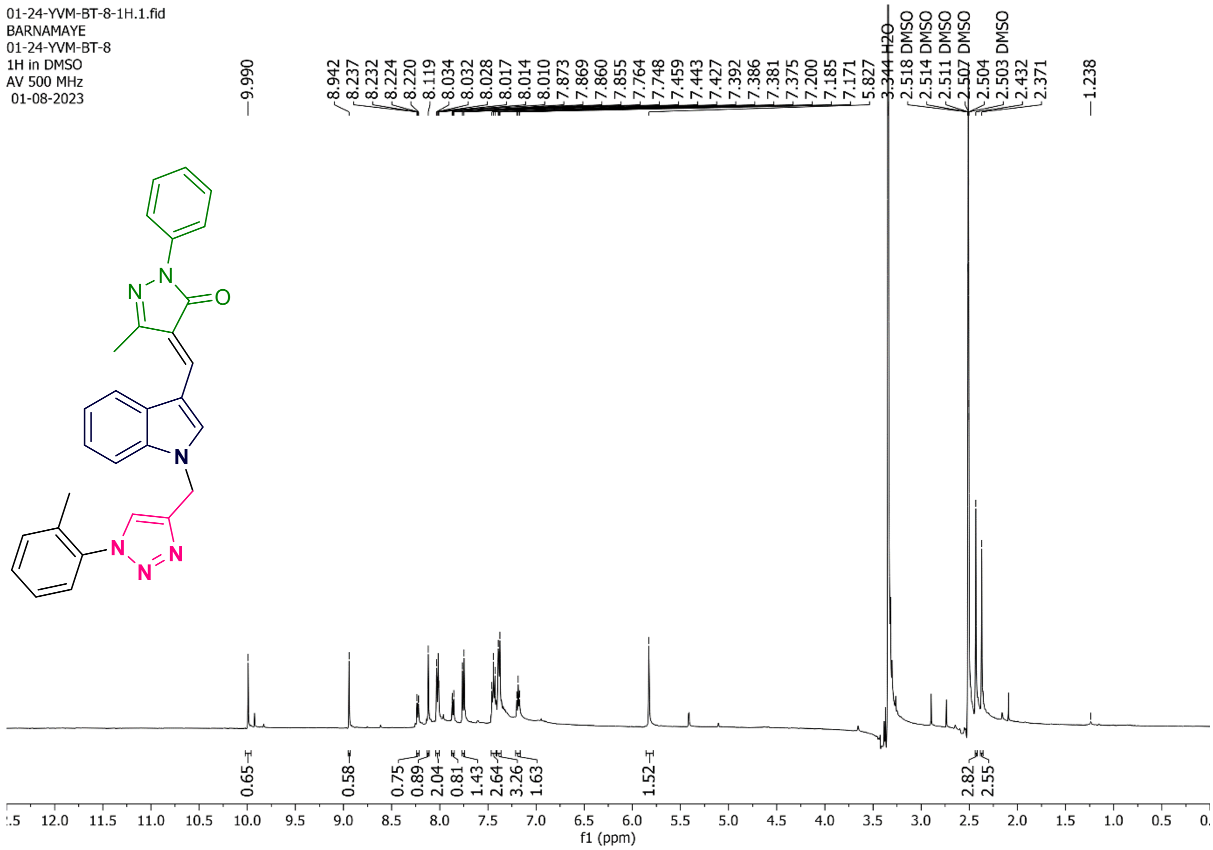

Figure 15. ^{1}H NMR spectrum of compound **5h** at 500 MHz in DMSO-d6

Figure 16. ^{13}C NMR spectrum of compound **5h** at 126 MHz in DMSO-d6

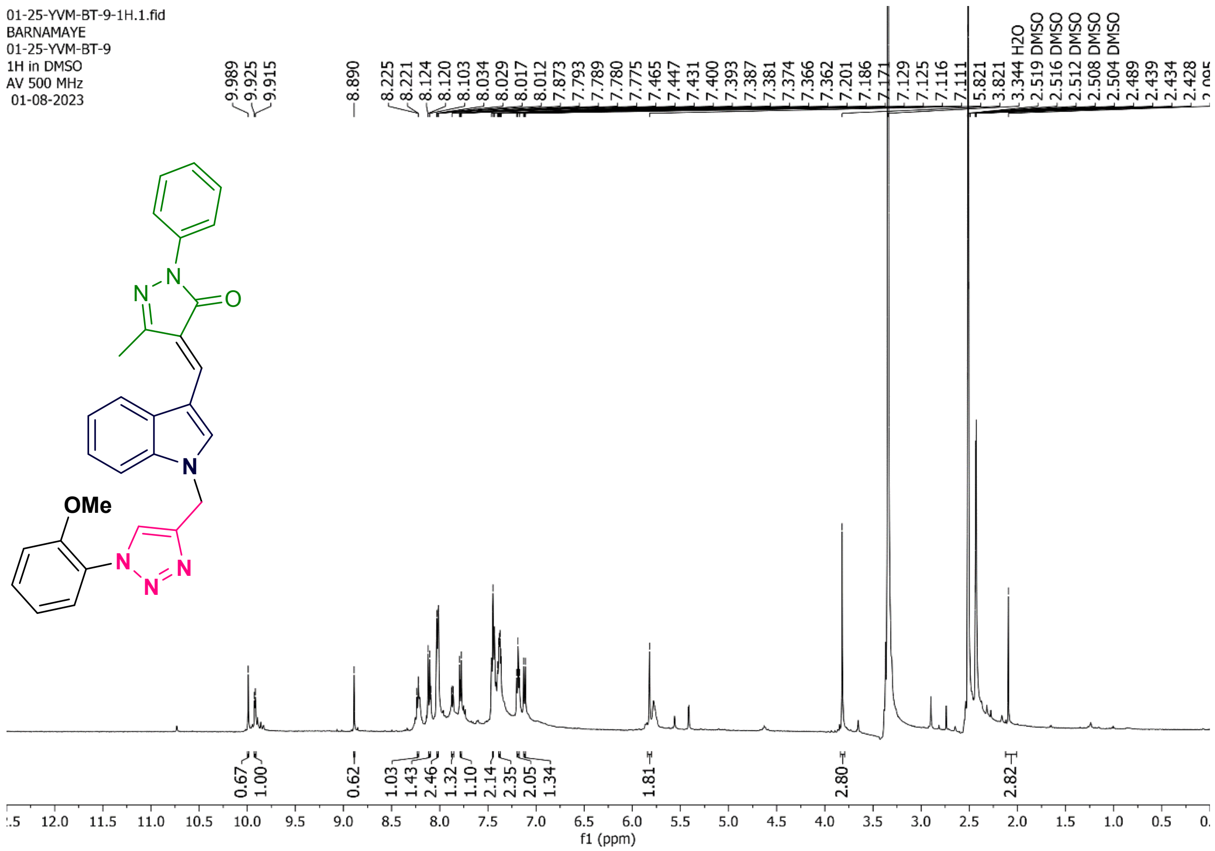

Figure 17. ^{1}H NMR spectrum of compound **5i** at 500 MHz in DMSO-d6

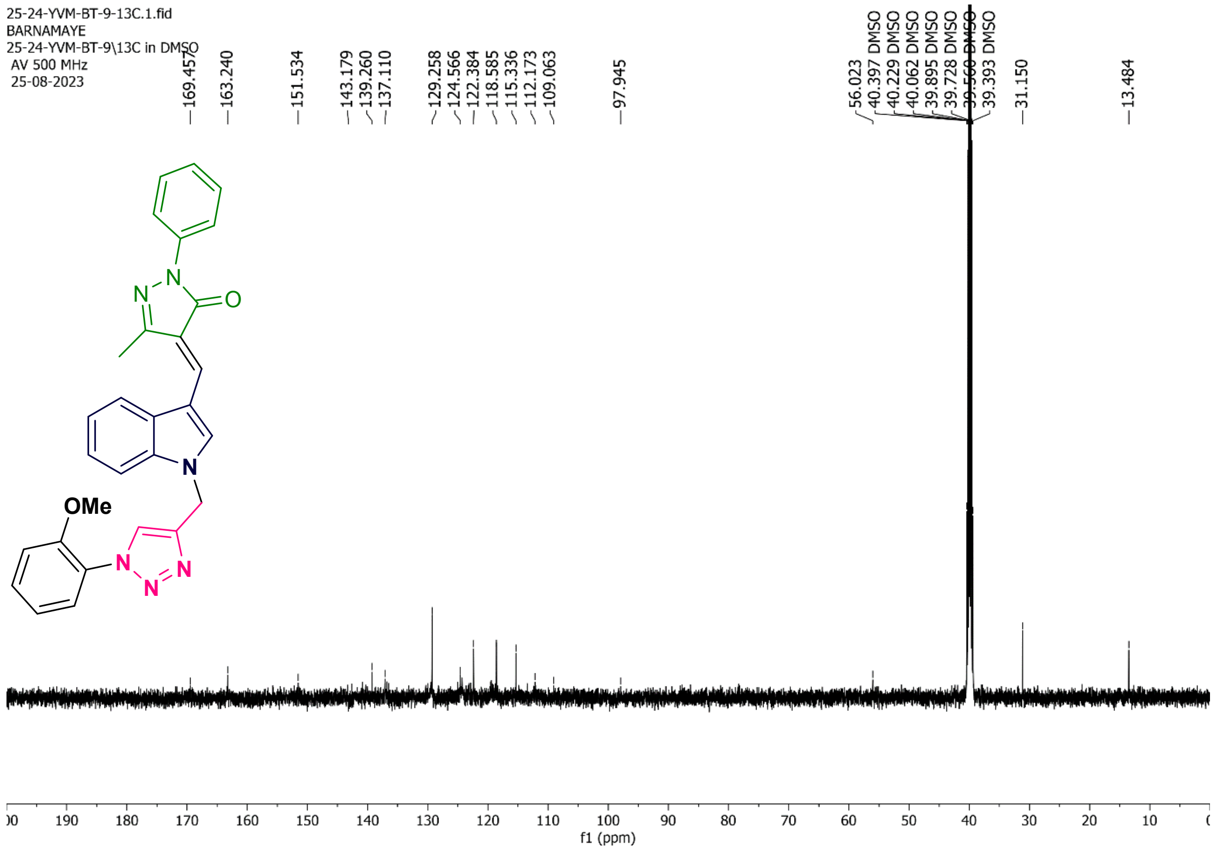

Figure 18. ^{13}C NMR spectrum of compound **5i** at 126 MHz in DMSO-d6

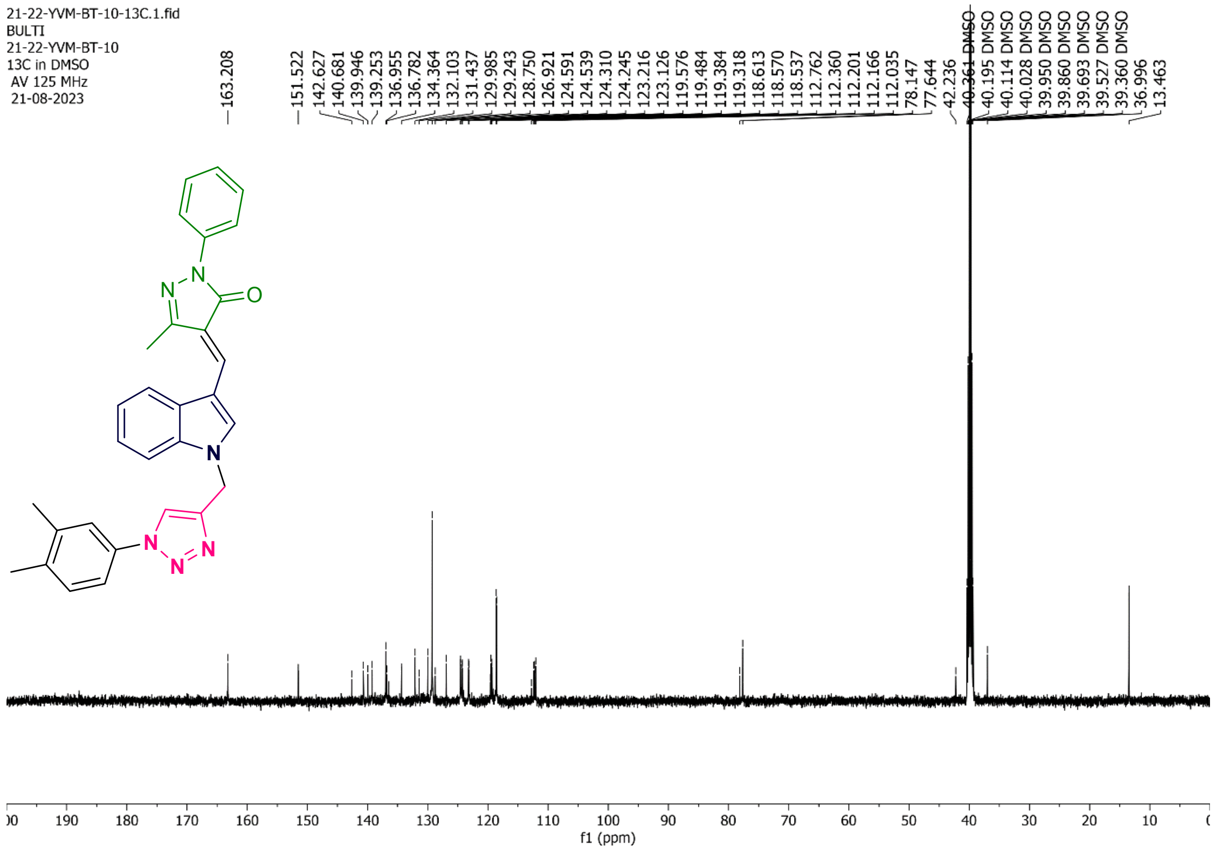

Figure 20. ^{13}C NMR spectrum of compound **5j** at 126 MHz in DMSO-d6

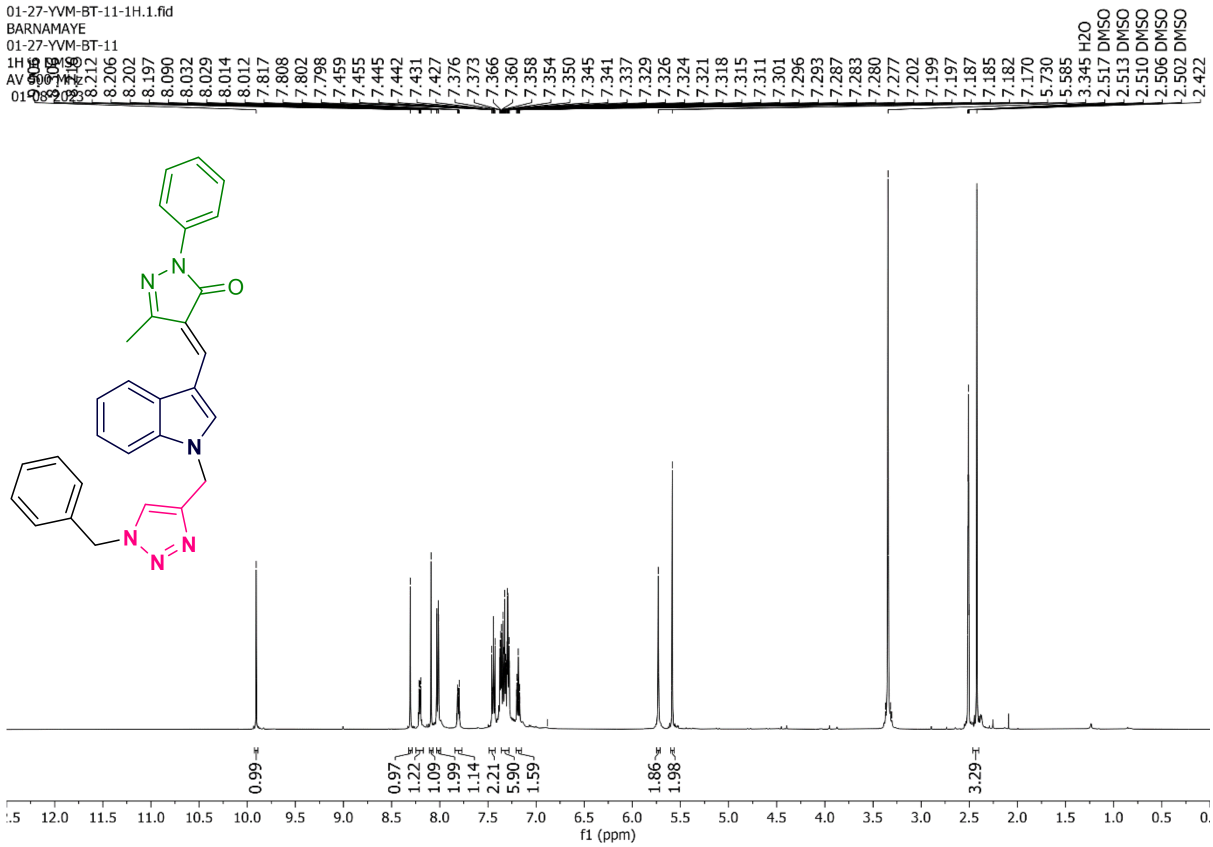

Figure 21. ^{1}H NMR spectrum of compound **5k** at 500 MHz in DMSO-d6

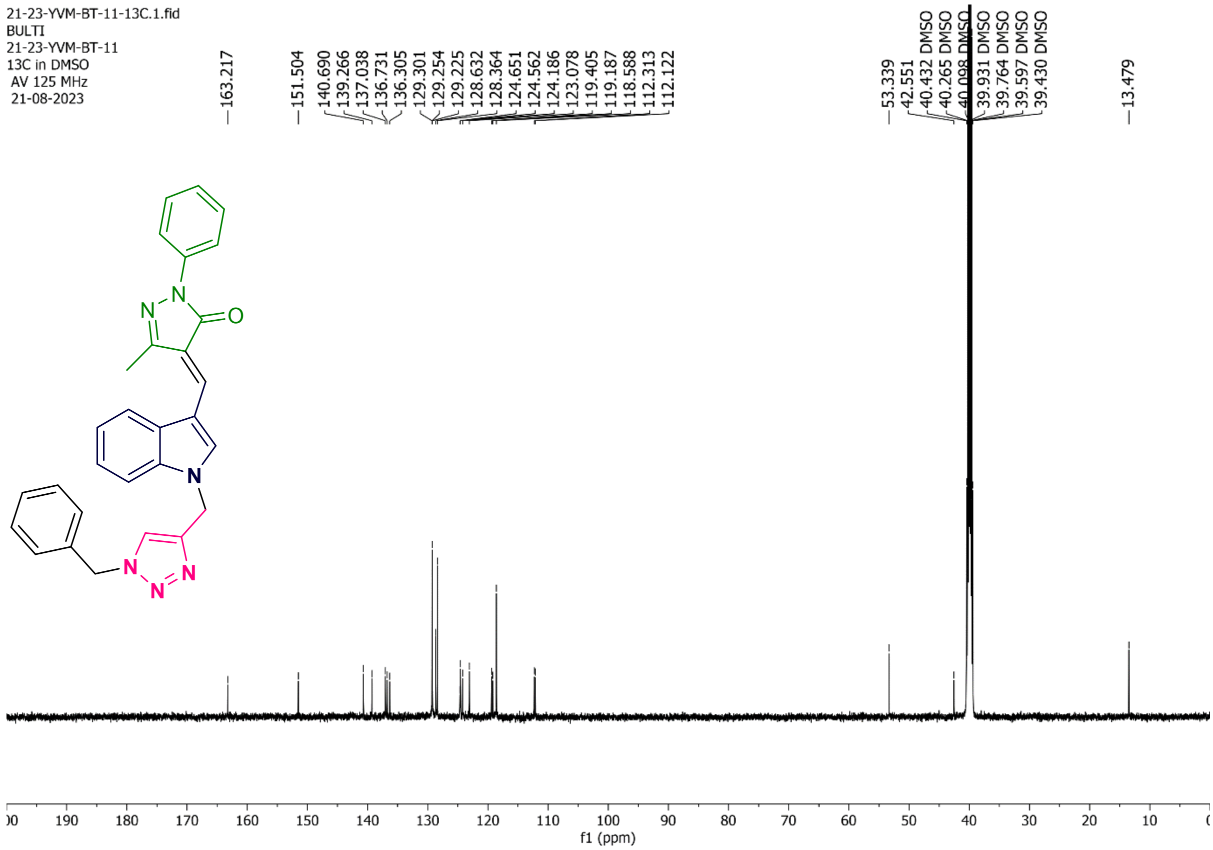

Figure 22. ^{13}C NMR spectrum of compound **5k** at 126 MHz in DMSO-d6

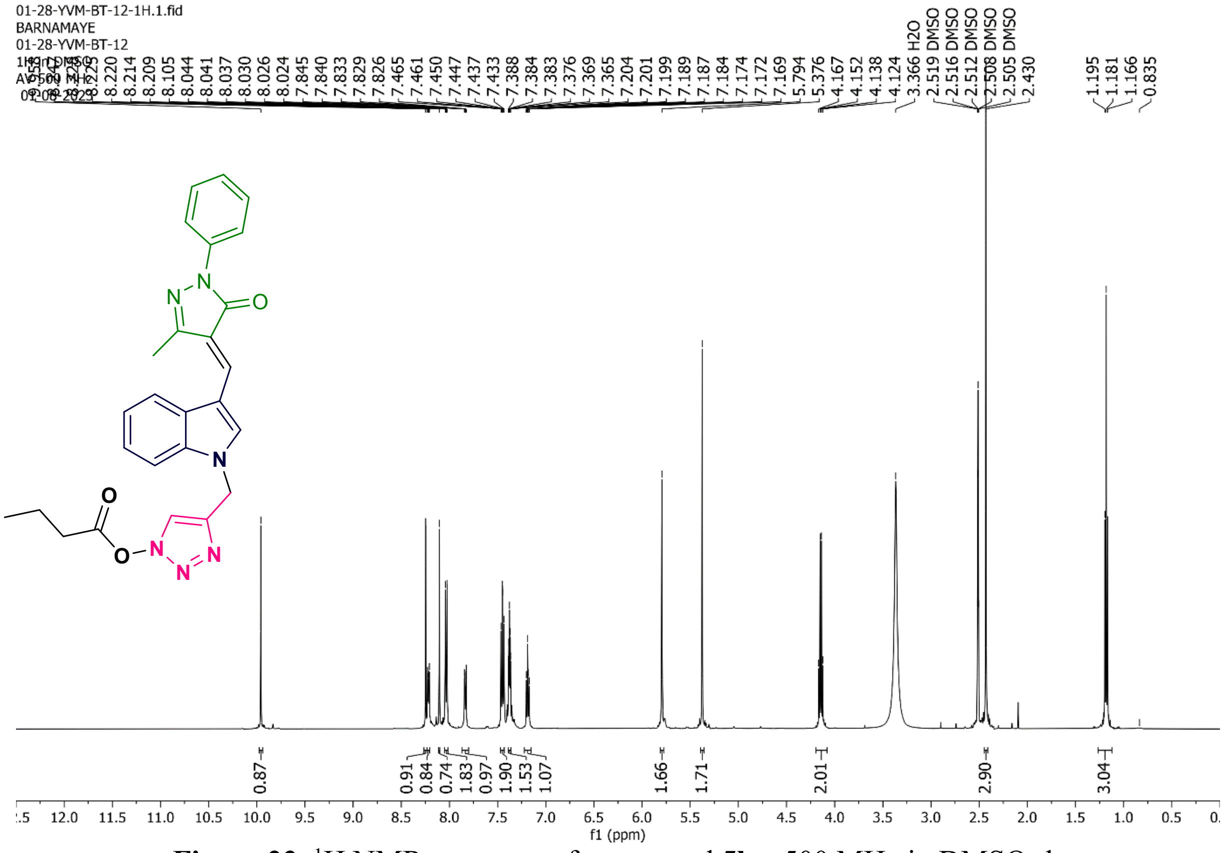

Figure 23. ^{1}H NMR spectrum of compound **5l** at 500 MHz in DMSO-d6

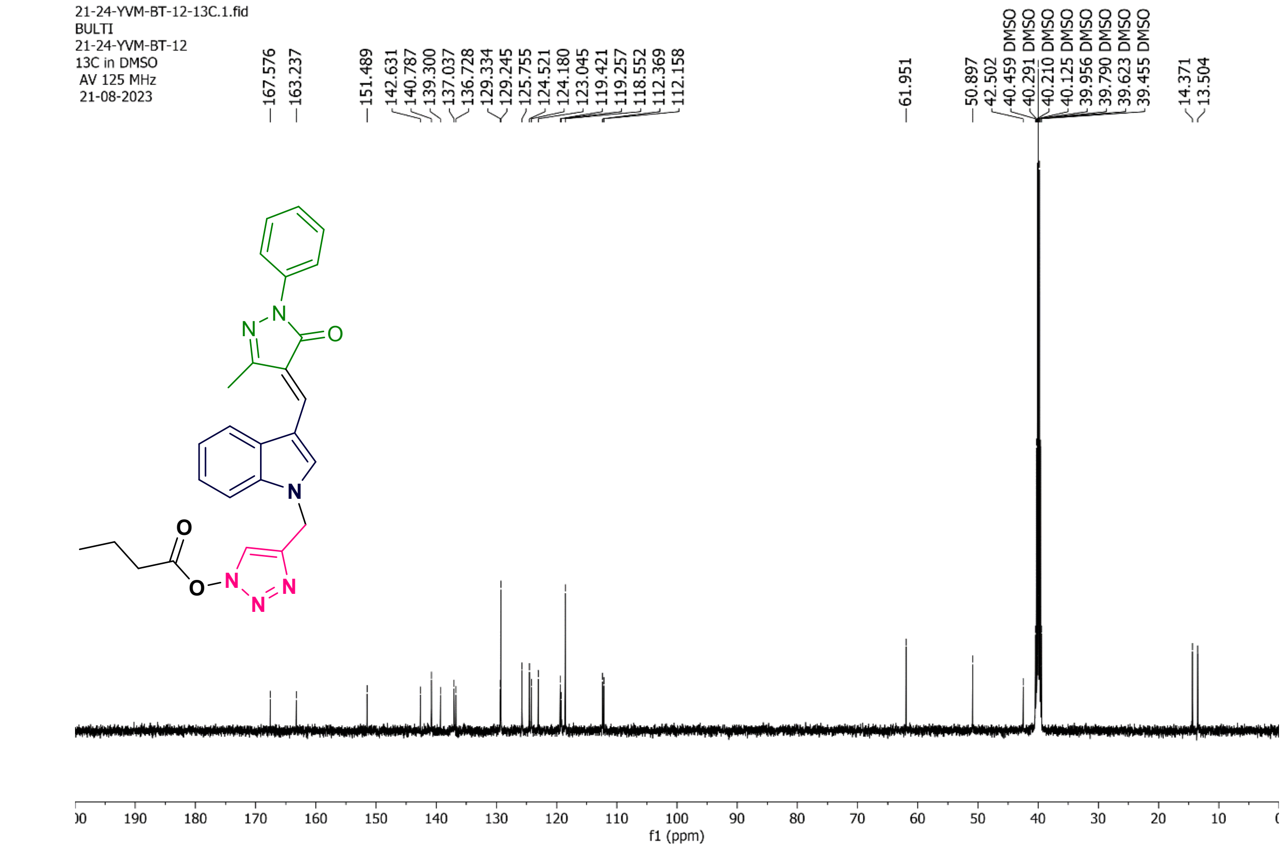

Figure 24. ^{13}C NMR spectrum of compound **5l** at 126 MHz in DMSO-d6

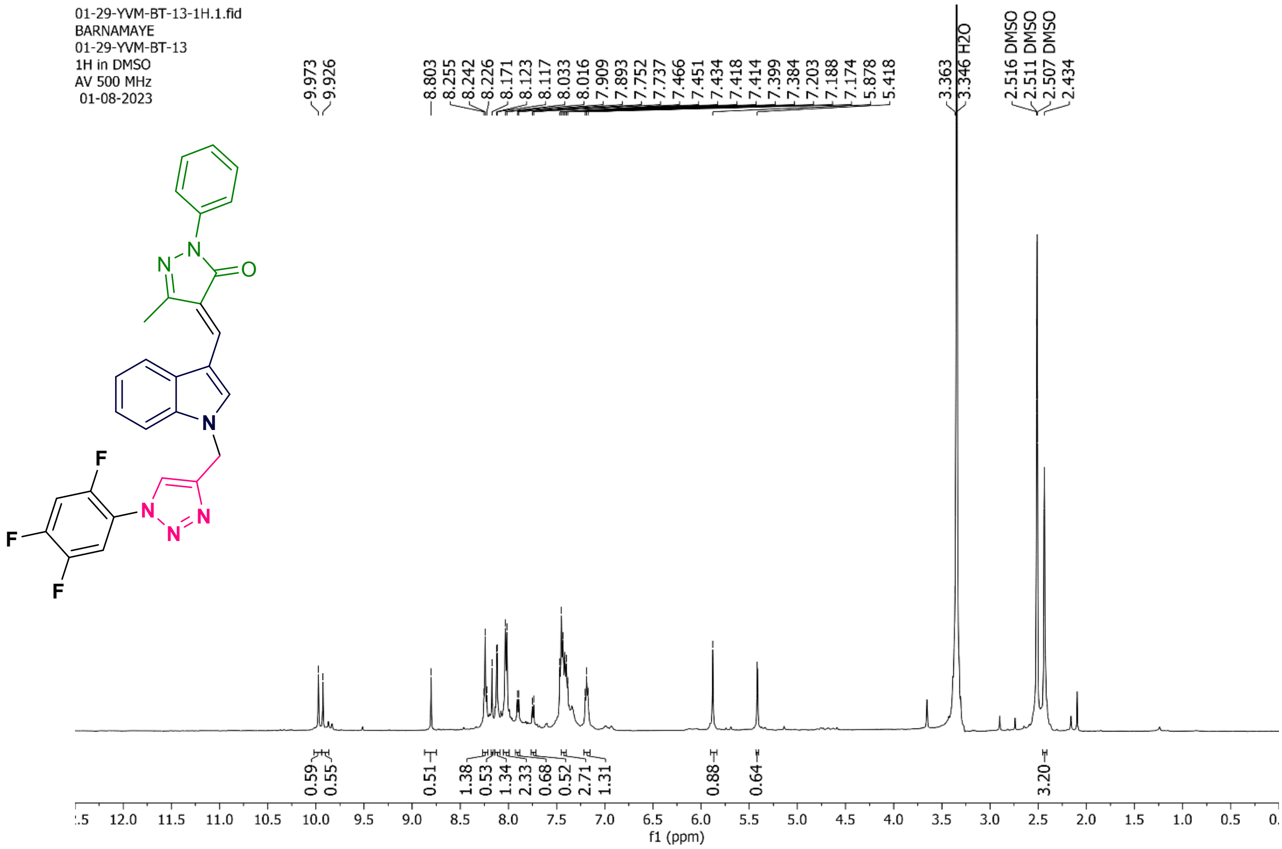

Figure 25. ^{1}H NMR spectrum of compound **5m** at 500 MHz in DMSO-d6

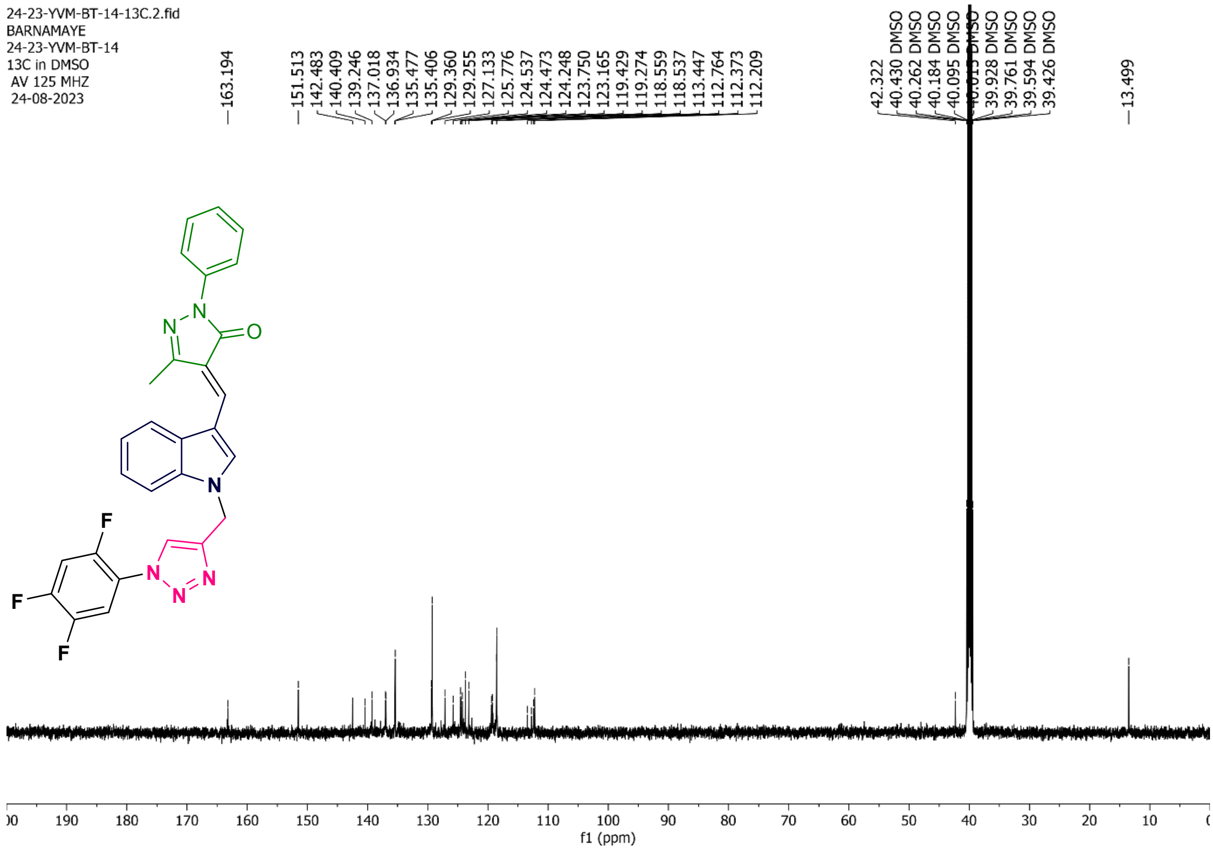

Figure 26. ^{13}C NMR spectrum of compound **5m** at 126 MHz in DMSO-d6

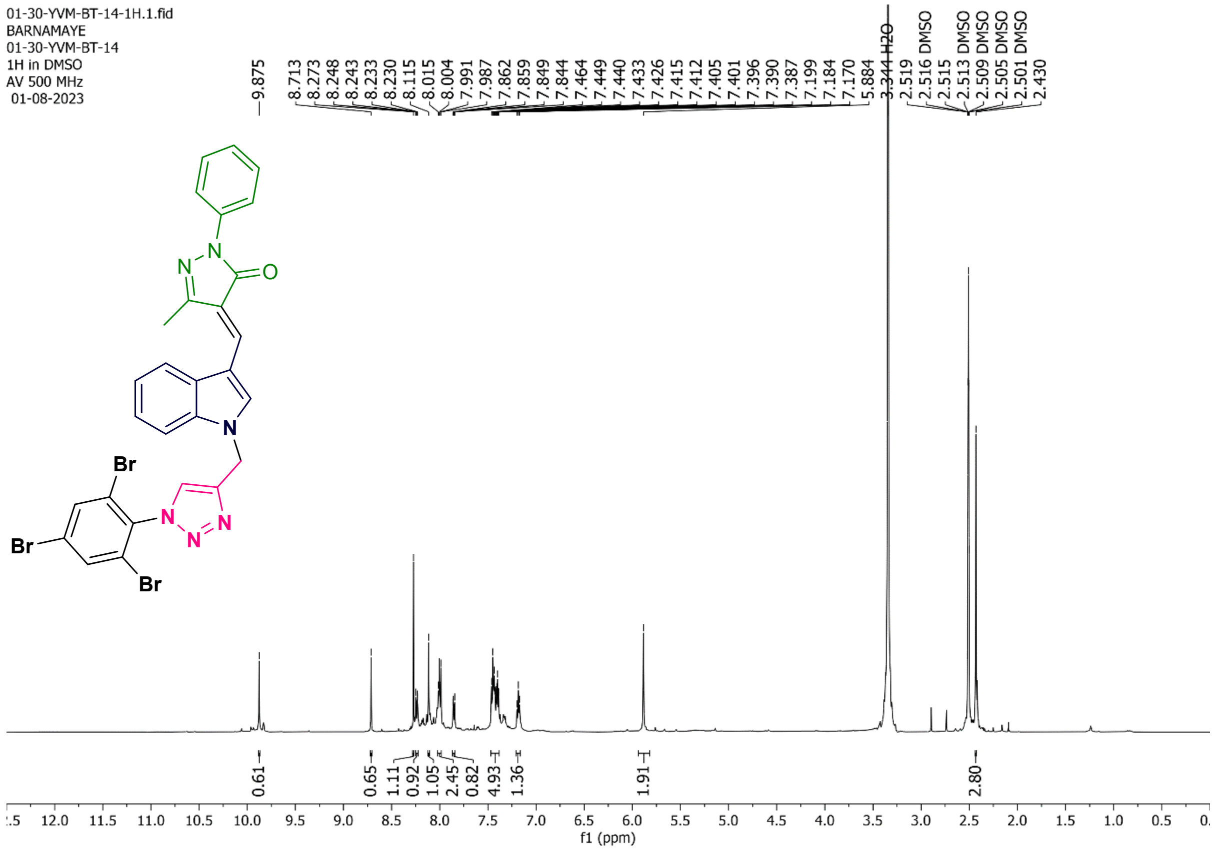

Figure 27. ^{1}H NMR spectrum of compound **5n** at 500 MHz in DMSO-d6

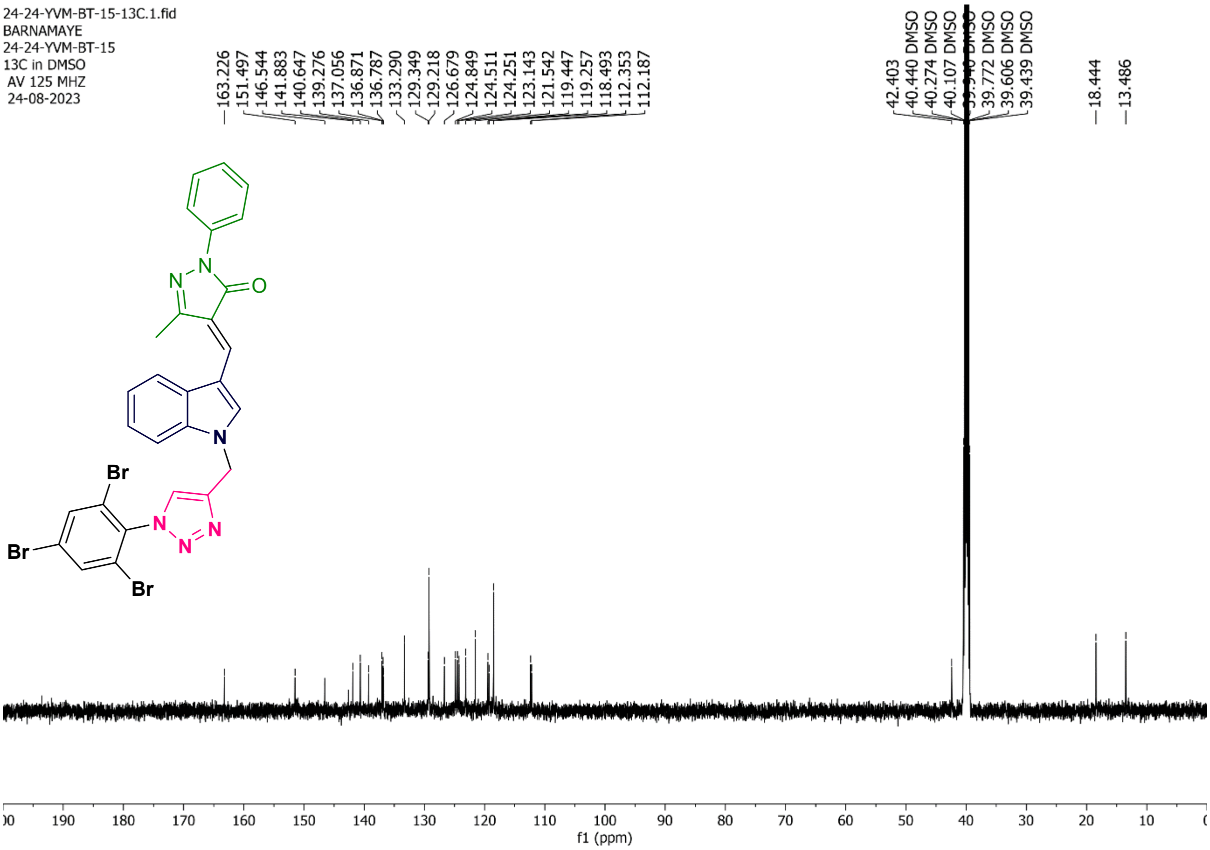

Figure 28. ^{13}C NMR spectrum of compound **5n** at 126 MHz in DMSO-d6

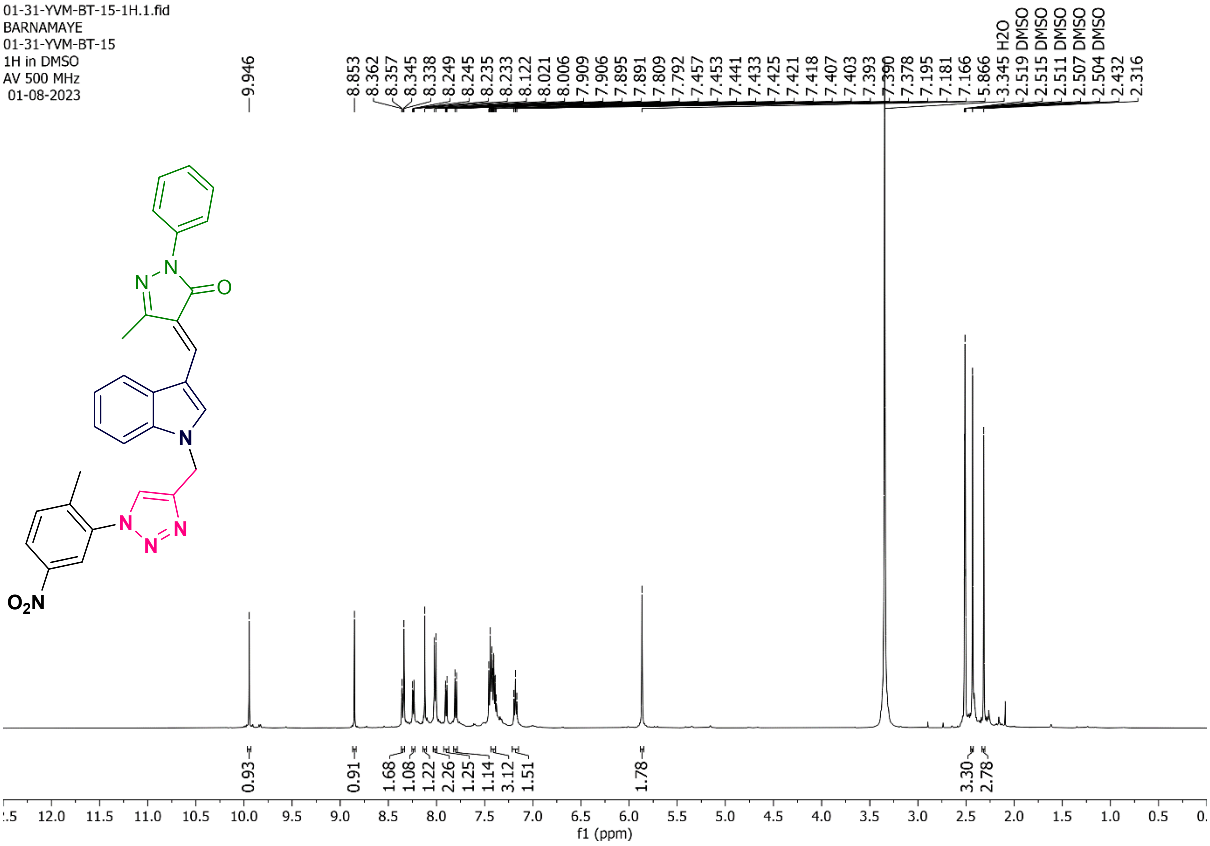

Figure 29. ^{1}H NMR spectrum of compound **5o** at 500 MHz in DMSO-d6

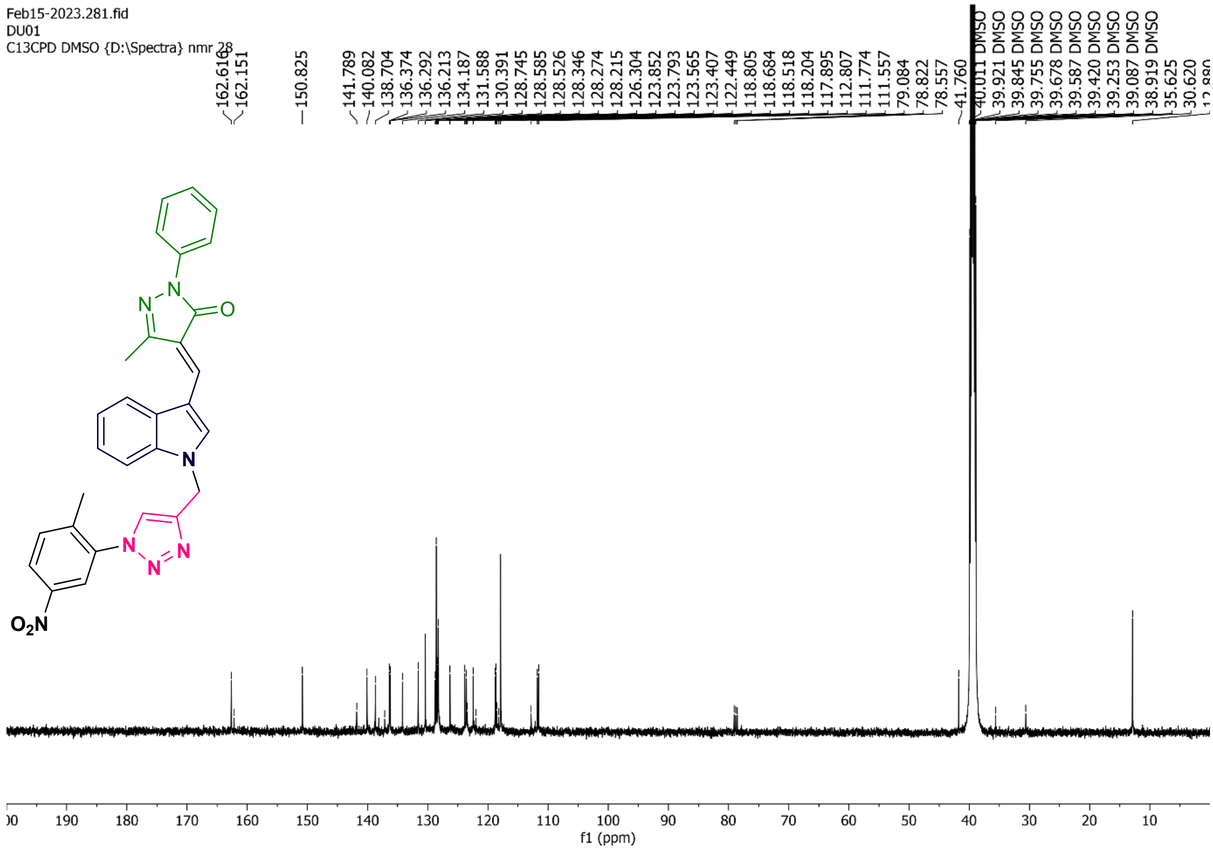

Figure 30. ^{13}C NMR spectrum of compound **5o** at 126 MHz in DMSO-d6

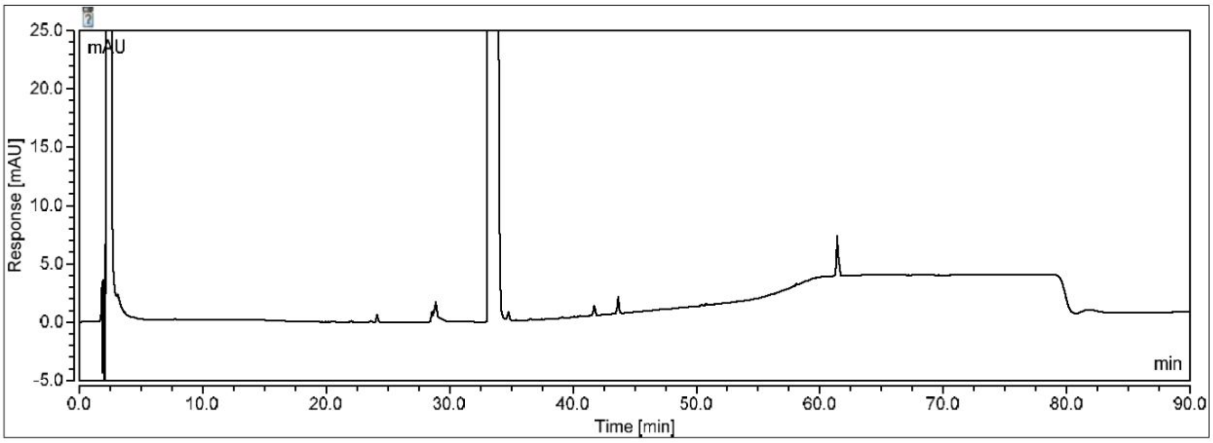

PEAK RESULT

No.	Ret.Time min	Peak Name	Area mAU*sec	Area %	R.R.T.
1	22.04	Unk	1.318	0.01	0.66
2	23.64	Unk	1.864	0.02	0.71
3	24.14	Unk	6.465	0.06	0.73
4	28.56	Unk	6.780	0.06	0.86
5	28.88	Unk	24.791	0.21	0.87
6	33.18	DB-1	11432.523	99.10	1.00
7	34.76	Unk	7.417	0.06	1.05
8	40.53	Unk	1.381	0.01	1.22
9	41.71	Unk	7.586	0.07	1.26
10	43.66	Unk	12.472	0.11	1.32
11	61.40	Unk	26.499	0.23	1.85
12	61.53	Unk	7.611	0.07	1.85
Total			11536.707	100.00	

HPLC data of **5a**

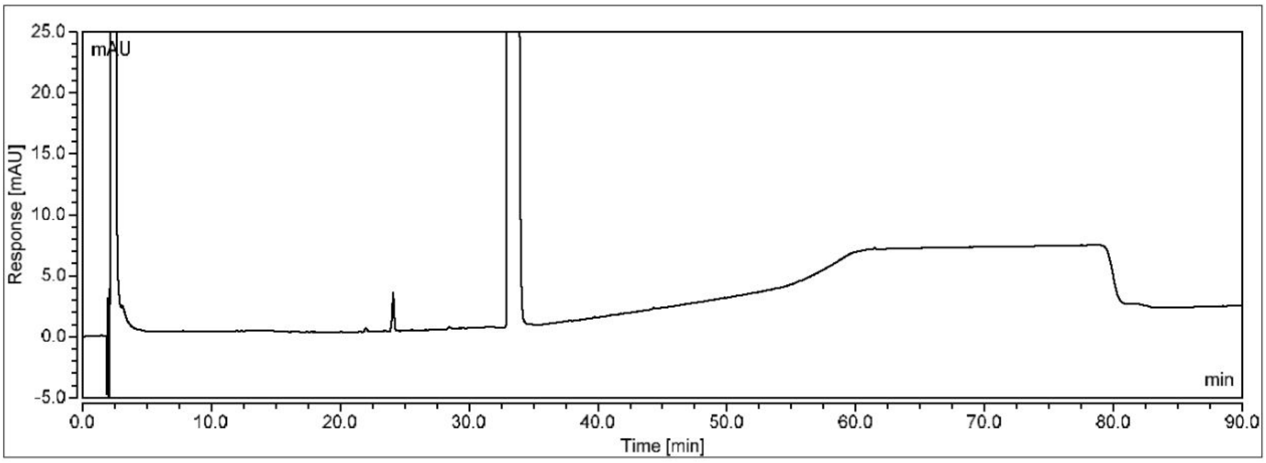

PEAK RESULT

No.	Ret.Time<br>min	Peak Name	Area<br>mAU*sec	Area<br>%	R.R.T.
1	22.01	Unk	4.010	0.03	0.67
2	23.47	Unk	0.718	0.01	0.71
3	24.10	Unk	29.149	0.22	0.73
4	28.49	Unk	2.115	0.02	0.86
5	33.04	DB-2	13433.480	99.69	1.00
6	34.79	Unk	1.486	0.01	1.05
7	37.79	Unk	0.937	0.01	1.14
8	41.03	Unk	0.642	0.00	1.24
9	41.74	Unk	0.925	0.01	1.26
10	44.31	Unk	0.938	0.01	1.34
11	61.43	Unk	1.170	0.01	1.86
Total			13475.568	100.00	

HPLC data of **5b**

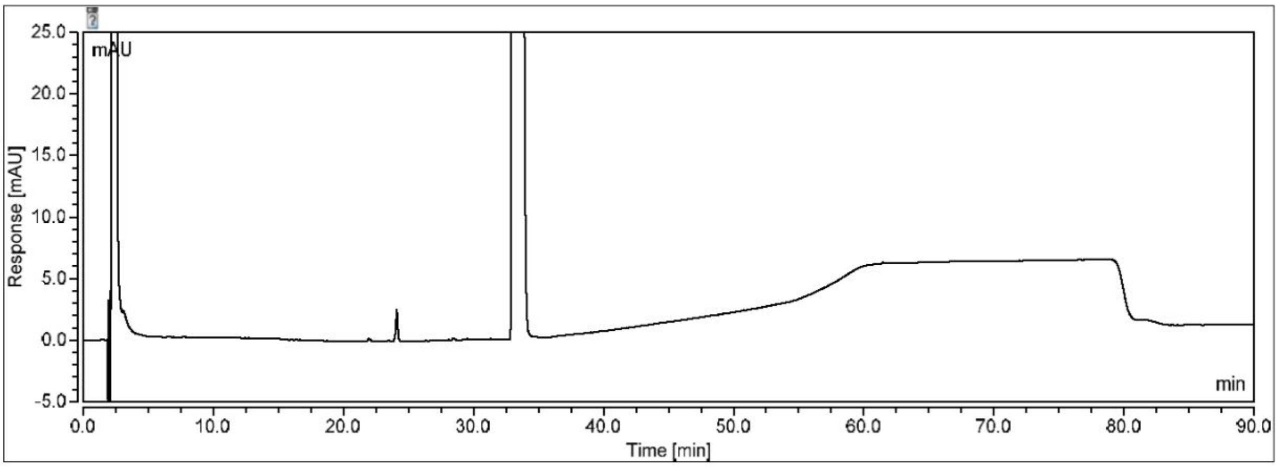

PEAK RESULT

No.	Ret.Time<br>min	Peak Name	Area<br>mAU*sec	Area<br>%	R.R.T.	Resolution<br>USP
1	22.02	Unk	2.977	0.02	0.67	n.a.
2	23.48	Unk	1.067	0.01	0.71	4.81
3	24.10	Unk	24.519	0.17	0.73	2.17
4	28.50	Unk	2.054	0.01	0.86	19.04
5	29.52	Unk	0.657	0.00	0.89	4.89
6	33.00	DB - 3	14780.204	99.77	1.00	6.11
7	34.76	Acetyl RESA-II	1.321	0.01	1.05	2.97
8	37.77	Unk	0.616	0.00	1.14	13.63
9	61.46	Unk	1.086	0.01	1.86	108.30
Total :			14814.500	100.00		

HPLC data of **5c**

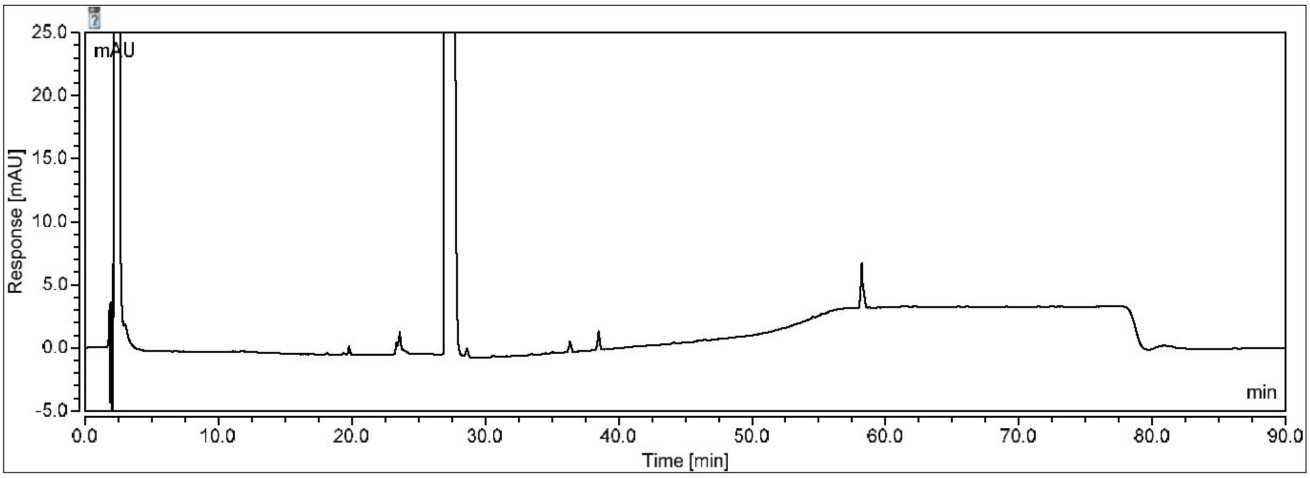

PEAK RESULT

No.	Ret.Time min	Peak Name	Area mAU*sec	Area %	R.R.T.
1	17.50	Unk	1.318	0.01	0.66
2	19.04	Unk	1.864	0.02	0.71
3	22.64	Unk	6.465	0.06	0.73
4	23.56	Unk	6.780	0.06	0.86
5	23.88	Unk	24.791	0.21	0.87
6	27.50	DB - 4	11432.523	99.10	1.00
7	29.76	Unk	7.417	0.06	1.05
8	30.53	Unk	1.381	0.01	1.22
9	31.71	Unk	7.586	0.07	1.26
10	33.66	Unk	12.472	0.11	1.32
11	56.40	Unk	26.499	0.23	1.85
12	61.53	Unk	7.611	0.07	1.85
Total			11536.707	100.00	

HPLC data of **5d**

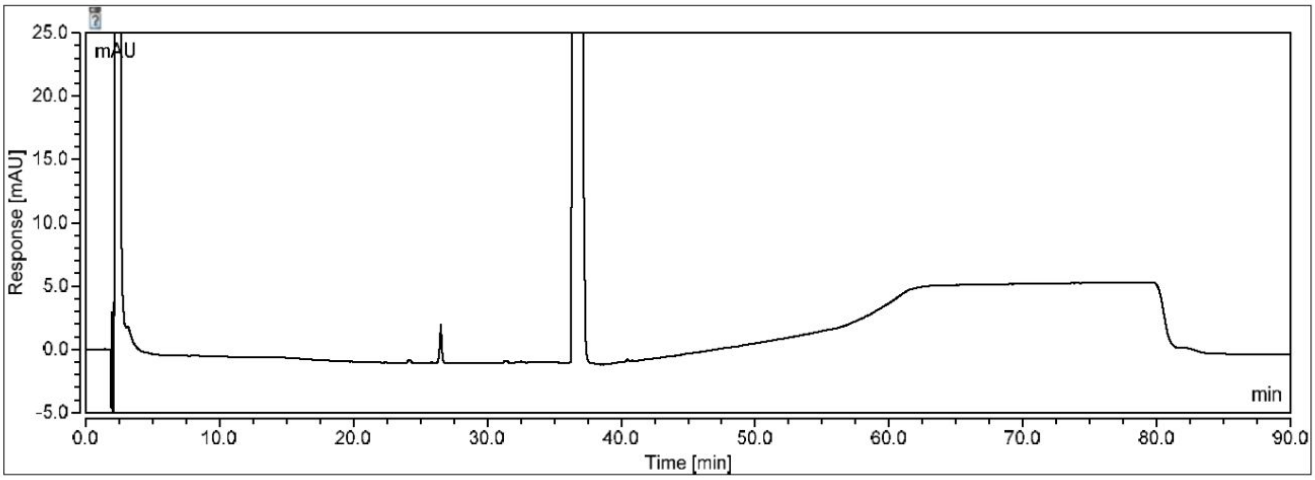

PEAK RESULT

No.	Ret.Time min	Peak Name	Area mAU*sec	Area %	R.R.T.	Resolution USP
1	24.03	Unk	3.479	0.03	0.67	n.a.
2	25.47	Unk	0.934	0.01	0.71	5.99
3	26.12	Unk	27.129	0.20	0.73	2.81
4	30.50	Unk	2.086	0.02	0.86	18.56
5	35.05	DB - 5	13449.162	99.74	1.00	8.04
6	39.43	Unk	1.820	0.01	1.13	7.67
Total :			13484.609	100.00		

HPLC data of **5e**

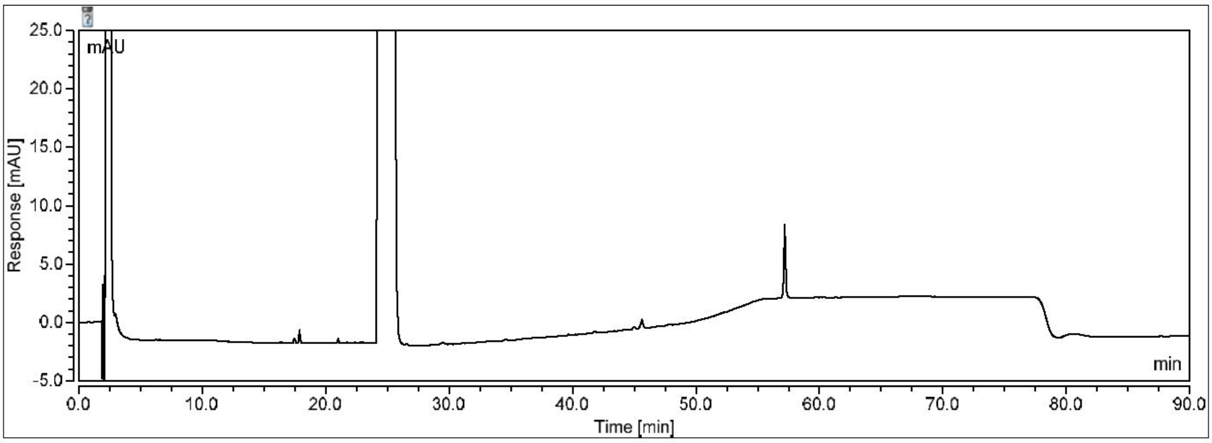

PEAK RESULT

No.	Ret.Time min	Peak Name	Area mAU*sec	Area %	R.R.T.	Resolution USP
1	16.58	Unk	5.674	0.04	0.71	n.a.
2	17.15	Unk	9.887	0.07	0.73	2.05
3	19.56	Unk	2.999	0.02	0.86	18.89
4	25.01	DB - 6	14192.124	99.42	1.00	7.99
5	26.78	Unk	2.301	0.02	1.05	2.71
6	28.37	Unk	3.129	0.02	1.13	7.01
7	31.74	Unk	1.231	0.01	1.26	13.81
8	38.04	Unk	1.779	0.01	1.45	27.09
9	40.82	Unk	2.235	0.02	1.54	12.22
10	51.36	Unk	7.824	0.05	1.55	2.14
11	57.44	Unk	45.674	0.32	1.86	41.24
Total :			14274.858	100.00		

HPLC data of **5f**
